# Contrasting Phylogeographic Patterns in *Lumnitzera* Mangroves Across the Indo-West Pacific

**DOI:** 10.3389/fpls.2021.637009

**Published:** 2021-06-23

**Authors:** Wuxia Guo, Achyut Kumar Banerjee, Haidan Wu, Wei Lun Ng, Hui Feng, Sitan Qiao, Ying Liu, Yelin Huang

**Affiliations:** ^1^Department of Bioengineering, Zhuhai Campus of Zunyi Medical University, Zhuhai, China; ^2^State Key Laboratory of Biocontrol and Guangdong Provincial Key Laboratory of Plant Resources, School of Life Sciences, Sun Yat-sen University, Guangzhou, China; ^3^China-ASEAN College of Marine Sciences, Xiamen University Malaysia, Sepang, Malaysia

**Keywords:** congeneric species, conservation, genetic differentiation, mangroves, population structure

## Abstract

Mangroves are ecologically important forest communities in tropical and subtropical coasts, the effective management of which requires understanding of their phylogeographic patterns. However, these patterns often vary among different species, even among ecologically similar taxa or congeneric species. Here, we investigated the levels and patterns of genetic variation within *Lumnitzera* consisting of two species (*L. racemosa* and *L. littorea*) with nearly sympatric ranges across the Indo-West Pacific (IWP) region by sequencing three chloroplast DNA regions (for both species) and genotyping 11 nuclear microsatellite loci (for *L. littorea*). Consistent with findings in studies on other mangrove species, we found that both *L. racemosa* and *L. littorea* showed relatively high genetic variation among populations but low genetic variation within populations. Haplotype network and genetic clustering analyses indicated two well-differentiated clades in both *L. racemosa* and *L. littorea*. The relationship between geographic and genetic distances and divergence time estimates of the haplotypes indicated that limited dispersal ability of the propagules, emergence of land barriers during ancient sea-level changes, and contemporary oceanic circulation pattern in the IWP influenced the current population structure of the two species. However, the position of genetic break was found to vary between the two species: in *L. racemosa*, strong divergence was observed between populations from the Indian Ocean and the Pacific Ocean possibly due to land barrier effect of the Malay Peninsula; in *L. littorea*, the phylogeographic pattern was created by a more eastward genetic break along the biogeographic barrier identified as the Huxley’s line. Overall, our findings strongly supported previous hypothesis of mangrove species divergence and revealed that the two *Lumnitzera* species have different phylogeographic patterns despite their close genetic relationship and similar current geographic distribution. The findings also provided references for the management of *Lumnitzera* mangroves, especially for the threatened *L. littorea*.

## Introduction

Approximately 70 woody plant species belonging to about 30 genera in 20 families are generally recognized as being mangroves (Duke, 2017). They are vital members of the intertidal wetland ecosystems and directly impact the welfare of coastal communities in the tropics and subtropics. Although adaptation to different habitats shapes their local distributional patterns, it is likely that a combination of current dispersal and historical perturbations has a predominant role in determining the overall distribution patterns of mangrove populations (Duke, 1995). However, mangroves are severely undermined by widespread habitat degradation and destruction to the point that they are predicted to be lost by the end of the century if the current practices continue (Duke et al., 2007). Thus, a better understanding of the current levels and patterns of genetic variation in mangrove populations, as well as their evolutionary histories, may provide useful information for the effective management of extant populations.

Many recent molecular studies in the Indo-West Pacific (IWP) region, one of the two mangrove diversity centers of the world (along with the Atlantic East Pacific; Guo et al., 2018c), have revealed that genetic discontinuities within marine species are formed by a variety of barriers to gene flow. For example, the Malay Peninsula has shaped genetic structure of marine organisms between the Indian and Pacific oceans (e.g., Timm and Kochzius, 2008; Adibah et al., 2015). Some other “cryptic barriers” in the West Pacific can also be found in the literature, e.g., the Wallace’s line (Duke, 2017) as well as its modified versions such as the Huxley’s line (Simpson, 1977). For the mangroves, recent studies have shown that the Malay Peninsula and/or ocean currents in the IWP have played important roles in shaping patterns of genetic variation (Tomizawa et al., 2017), even among members within major mangrove genera such as *Rhizophora* (Ng et al., 2015; Wee et al., 2015) and *Xylocarpus* (Guo et al., 2018b). Such differences in phylogeographic patterns among closely related species have often been attributed to subtle differences in dispersal abilities and/or the evolutionary histories of individual species (Ng et al., 2015). Therefore, given the polyphyletic origin of mangroves (Duke, 1995), it is important to explore the genetic characteristics of mangrove species having different life histories.

*Lumnitzera* (Family: Combretaceae), commonly known as the black mangrove, is a non-viviparous mangrove genus distributed in the IWP region (Tomlinson, 2016). The genus comprises of two species, *Lumnitzera racemosa* and *Lumnitzera littorea*, which differ strikingly in the color of their flowers, with *L. racemosa* having white flowers and *L. littorea* having red flowers. There is also a rare, reportedly sterile, hybrid *L.* × *rosea*, with pink flowers and several other intermediate characters (Tomlinson, 2016). The geographic distribution of *L. littorea* largely overlaps with *L. racemosa* from India through Southeast Asia, north to China, and south to Australia. The two species differ in habitat, with *L. racemosa* preferring areas with higher salinity, while *L. littorea* occurs at the landward margin of mangrove areas with less saline and infrequent inundation; both species are seldom found growing together (Tomlinson, 2016), a pattern being similar to other mangrove taxa (e.g., ecological separation of *Pelliciera rhizophorae* and *P. benthamii* in the Atlantic East Pacific, Duke, 2020). Previous studies have shown similar genetic divergence patterns for the two species (Su et al., 2006, 2007), but these investigations suffer from a small sample size and limitations in the genetic data due to the dominant nature of the markers used. A recent study using multiple nuclear genes suggested the possible role of the Malay Peninsula in causing pronounced genetic differentiation (even compared to other mangrove species) between *L. racemosa* populations on both sides of the peninsula (Li et al., 2016). As *L. racemosa* and *L. littorea* have similar distribution ranges, we hypothesized that the barriers in the region would affect the two species in similar ways, leading to similar phylogeographic patterns such as those found between *Ceriops tagal* and *Ceriops decandra* (Huang et al., 2008) as well as between *Sonneratia caseolaris* and *Sonneratia alba* (Yang et al., 2016).

In this study, we aimed to address the following questions: (1) What is the pattern of genetic variation across the distribution of *L. littorea*? (2) Does the Malay Peninsula also serve as a land barrier in *L. littorea*, as demonstrated in *L. racemosa*? (3) Are there any other cryptic barriers that could have affected the current distribution and patterns of genetic variation of *L. littorea* and *L. racemosa*? We start by inferring the period of divergence between *Lumnitzera* and its closest relative, and then compare the genetic structures of its member species across their distributions using maternally inherited chloroplast DNA (cpDNA). Further, a set of 11 bi-parentally inherited nuclear DNA markers are also used to infer fine-scale genetic patterns in *L. littorea*, especially contemporary gene flow mirrored by more recent demographic history.

## Materials and Methods

### Population Sampling and DNA Extraction

**Figure 1:**
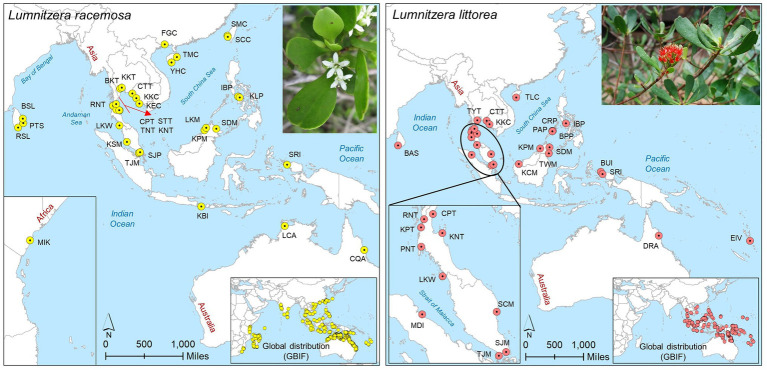
Sampling locations of *L. racemosa* and *L. littorea* in the Indo-West Pacific (IWP); Abbreviations of the populations have been given in Supplementary Table S1 (inset up – species picture; inset below – global distribution of the species as retrieved from GBIF: https://doi.org/10.15468/dl.6z9u9f for *L. racemosa* and https://doi.org/10.15468/dl.8eujdv for *L. littorea*).

Leaf samples were collected from 32 populations (385 individuals) of *L. racemosa* and 27 populations (329 individuals) of *L. littorea* across the IWP region (Figure 1; Supplementary Table S1). Only young and healthy leaves were collected from mature trees over 2 m tall, and these individuals were spaced at least 10 m apart. Leaf samples were stored with silica gel until use. Total genomic DNA was extracted from dried leaf material using the CTAB method (Doyle and Doyle, 1990).

### Chloroplast DNA Sequencing and Microsatellite Genotyping

For cpDNA sequences analysis, after preliminary screening of 31 available primer pairs at different chloroplast regions, three cpDNA intergenic spacer regions, *trn*T*-trn*L, *trn*G*-trn*S, and *atp*B*-rbc*L, were found to produce single clear amplified bands and contained the most polymorphic sites (Supplementary Table S2). Therefore, these cpDNA regions were sequenced for all *L. littorea* and *L. racemosa* samples. PCR was conducted in 30 μl reactions with 30 ng of template DNA, 1 × PCR buffer, 1 mM of dNTPs, 1.5 μl of each primer, and 1.5 U *Taq* DNA polymerase (TransGen Biotech). Thermocycling conditions were: initial denaturation at 94°C for 4 min, followed by 30 cycles of 94°C for 40 s, 55–60°C for 1 min and 72°C for 1 min, and a final extension of 72°C for 8 min. The amplified fragments were checked through agarose gel electrophoresis and sequenced on an ABI 3730XL DNA Analyzer (Applied Biosystems).

In this study, samples of *L. littorea* were only considered for genotyping at microsatellite loci since detailed genetic structure of *L. racemosa* in the IWP has previously been studied using multiple nuclear genes (Li et al., 2016). For nuclear microsatellite (hereafter, nSSR) analysis, 50 primer pairs that amplify nuclear microsatellite regions were designed from leaf transcriptome data of *L. littorea* (unpublished data). Among these, 11 loci that showed stable amplification and high levels of polymorphism were selected for subsequent genotyping (Supplementary Table S2). PCR was conducted in 20 μl reactions with 20 ng of template DNA, 1 × PCR buffer, 1 mM of dNTPs, 1.0 μl of each primer, and 1 U of *Taq* DNA polymerase (TransGen Biotech). Thermocycling conditions were: initial denaturation at 94°C for 4 min, followed by 30 cycles of 94°C for 40 s, 53°C for 1 min and 72°C for 1 min, and a final extension of 72°C for 10 min. The amplified fragments were checked through agarose gel electrophoresis and genotyped on a Fragment Analyzer Automated CE System (Advanced Analytical Technologies) following the manufacturer’s protocol. Raw data were exported, and the number of alleles and allele sizes per locus were called using ProSize ver. 2.0 (Agilent). The amplified microsatellite sequences were also subjected to DNA sequencing on an ABI 3730XL DNA Analyzer (Applied Biosystems) for verification. Individual samples of eight populations could not be amplified successfully, therefore, a subset (i.e., 258 individuals) from 19 populations of *L. littorea* were genotyped at these loci (Supplementary Table S1). Due to small sample sizes (less than three individuals), two populations, i.e., BAS and IBP, could not be included in nSSR data-based analyses (except STRUCTURE and BARRIER analyses).

### Phylogenetic Analysis

The chloroplast sequence data were assembled and edited using SeqMan ver. 7.1.0 (DNAStar), aligned using CLUSTAL X ver. 1.83 (Thompson et al., 1997) and checked manually. The three cpDNA fragments, with consistent signal detected by incongruence length difference test (Farris et al., 1995), were concatenated for subsequent downstream analyses. Chloroplast haplotypes were identified and distinguished using DnaSP ver. 5.10 (Librado and Rozas, 2009). The geographical distribution of the haplotypes was plotted on a map using GenGIS ver. 2.2.2 (Parks et al., 2009), and their genealogical relationships were determined using the median-joining method in NETWORK ver. 5.0.0.1 (Bandelt et al., 1999). Continuous indels were treated as a single mutational event in the analysis.

Phylogenetic trees were constructed using MRBAYES ver. 3.2.7 (Ronquist and Huelsenbeck, 2003) and RAxML-NG ver. 0.9.0 (Kozlov et al., 2019), with *Laguncularia racemosa* as the outgroup, following Berger et al. (2016). The best-fit model of nucleotide substitution was inferred using the Akaike Information Criterion (AIC) implemented in the program MODELTEST ver. 3.8 (Posada and Crandall, 1998). The time-calibrated phylogeny among the two species were further estimated with BEAST ver. 2.5 (Bouckaert et al., 2019), incorporating a lognormal relaxed molecular clock, a Yule speciation process, and the GTR+I+G nucleotide substitution model as selected by MODELTEST. Two independent Markov coupled Markov (MCMC) chains of 3 × 10^7^ generations were performed and sampled every 3,000 generations with the first 10% of samples discarded as burn-in. TRACER ver. 1.6 (Rambaut et al., 2018) was used to examine the convergence of chain to the stationary distribution, and the effective sample size (ESS) of the posterior probability for each parameter was verified to be higher than 200 (except for “gammaShape” for which ESS was 180). The results were then summarized using TreeAnnotator ver. 2.4.3 (Bouckaert et al., 2014) and visualized using FigTree ver. 1.4. As there are no direct fossil records that could be used to calibrate the studied taxa, we first constructed a phylogenetic tree of the family Combretaceae based on transcriptomic data (ongoing work and unpublished data) and calibrated it with the fossil records retrieved from Berger et al. (2016).

### Phylogeographic Analysis

#### Genetic Diversity

Using the cpDNA data in DNASP ver. 5.10, the haplotype diversity (Hd) and nucleotide diversity (π) were calculated according to Nei (1987) at both the population (H_S_, π_s_) and species (H_T_, π_T_) levels. Average gene diversity within populations (H_S_) and total gene diversity (H_T_) were estimated for all populations using PERMUT ver. 1.0 (Pons and Petit, 1996). The coefficients of differentiation (G_ST_, N_ST_) for each species were calculated and compared through a permutation test with 1,000 permutations to evaluate the presence of phylogeographic structure using the same program.

The nSSR data for each locus and population were first checked for presence of null alleles and potential stuttering using MICRO-CHECKER ver. 2.2.3 (Van Oosterhout et al., 2004). Deviations from Hardy-Weinberg equilibrium (HWE) were tested for each locus and population in GENEPOP ver. 4.7.2 (Rousset, 2008). To avoid the uncertainty in the asymptotic methods (e.g., *χ*^2^-test) generated due to small sample size and/or involvement of rare alleles (Engels, 2009), we used exact Hardy-Weinberg (HW) test as implemented in GENEPOP. We tested the significance of heterozygote excess and deficiency for each population (at *p* = 0.05) using the score test (*U*-test) for which Markov chain algorithm was employed with 10,000 dememorization steps and the algorithm was run for 20 batches with 10,000 iterations per batch. To estimate the level of genetic diversity within the populations, the observed number of alleles (N_A_), effective number of alleles (N_E_), Shannon’s information index (I), and observed (H_O_) and expected (H_E_) heterozygosity were calculated using POPGENE ver. 1.32 (Yeh et al., 1997) whereas allelic richness (A_R_) was computed using FSTAT ver. 2.9.3 (Goudet, 2002). Inbreeding coefficient (F_IS_) for each population was estimated using GENEPOP whereas the percentage of polymorphic loci (PPB) were calculated with GENALEX ver. 6.5 (Peakall and Smouse, 2012).

#### Genetic Differentiation and Population Structure

To estimate population differentiation based on cpDNA sequence data, Hedrick’s G^/^_ST_ (standardized G_ST_; Hedrick, 2005) was estimated using the mmod package (Winter, 2012) in R. Using the nSSR data, pairwise population differentiation (F_ST_) values were first estimated using FSTAT and was further standardized (F^/^_ST_) using the RecodeData ver. 0.1 (Meirmans, 2006). These standardized measures of genetic differentiation were used to identify the biogeographical boundaries and isolation-by-distance (IBD) pattern between the populations. To identify the biogeographical boundaries exhibiting the largest genetic discontinuities between population pairs, barrier analysis was performed using BARRIER ver. 2.2 (Manni et al., 2004). To detect the IBD pattern, relationship between pairwise genetic distances and geographic distances (log-transformed values) was examined using Mantel tests (Mantel, 1967), as implemented in GENALEX, with 1,000 random permutations. The standardized measures of genetic differentiation were used in principal coordinate analysis (PCoA) in GenAlEx ver. 6.5.

Population structure of the species was evaluated with and without location information of the individual populations. Using the cpDNA sequence data, we first used the Bayesian Analysis of Population Structure (BAPS) version 6.0 (Corander et al., 2008) to group the individuals under subpopulations. For BAPS, we used “clustering with linked loci” genetic mixture analysis and the upper bound K values (i.e., the number of clusters) were set to 10. The number of iterations, the reference individuals, and the number of iterations were set to 100, 200, and 20, respectively. The optimal number of clusters was obtained by comparing the posterior probabilities of the pre-specified clusters. To evaluate the geographic influence on population structure, we used the spatial analysis of molecular variance (AMOVA; SAMOVA; Dupanloup et al., 2002) algorithm implemented in SPADS ver. 1.0 (Dellicour and Mardulyn, 2014). We considered models with putative numbers of populations (K) ranging from 1 to 10, and for each K, we used 10,000 simulations of the annealing process for each of the 100 repeated runs. The *K* value for which the largest genetic differentiation (F_CT_) value was obtained, was identified as the optimum grouping of populations.

Using the nSSR data of *L. littorea*, genetic clusters were determined using a Bayesian model-based clustering method implemented in STRUCTURE ver. 2.3.4 (Evanno et al., 2005). To obtain the optimal number of subpopulations (K), 20 runs were performed for each K (*K* = 1–10) under the admixture model and correlated allele frequency model without *a priori* information of the geographical information of the populations. Each run consisted of 1,000,000 replicates of MCMC after a burn-in of 1,000,000 replicates. The optimal K was determined using the ΔK parameter according to Evanno et al. (2005). To integrate the spatial information in determining the genetic clusters, we used a spatially explicit Bayesian clustering algorithm in the program GENELAND ver. 4.0.7 (Guillot et al., 2008) in R. For each of the 19 populations, we ran the Markov Chain Monte-Carlo (MCMC) simulations for 100,000 iterations under the spatial model with the assumption of uncorrelated allele frequencies between populations and each of the 1000th iteration was saved. These MCMC outputs were post-processed with a horizontal and vertical discretization of the study area in 100 pixels and a burn-in of 200 saved iterations to visualize the posterior distribution and membership probability of these populations.

Analysis of molecular variance was performed using ARLEQUIN ver. 3.5 (Excoffier et al., 1992) to evaluate the hierarchical partitioning of genetic variation among population groups (obtained from analyzing cpDNA and nSSR data, see Results), among populations within groups, and within populations, with 1,000 permutations to test the significance of the results.

#### Demographic History

**Figure 2:**
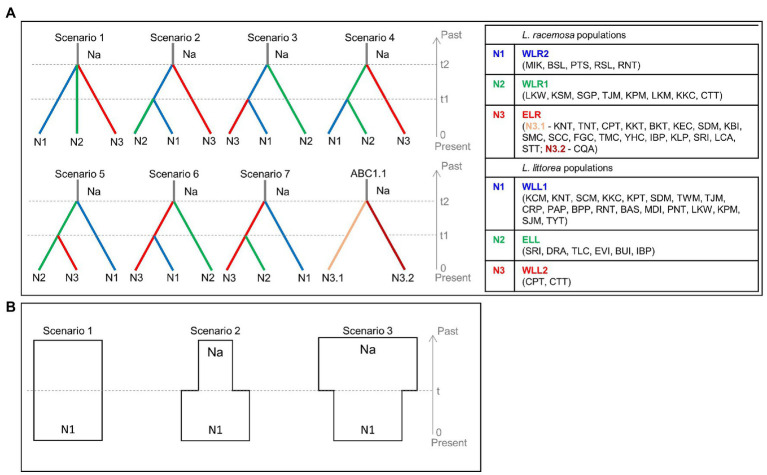
Conceptual models to assess the demographic history of *L. racemosa* and *L. littorea* through the approximate Bayesian computation (ABC) approach – **(A)** the seven scenarios tested to estimate the divergence time between the three population groups (ABC1), ABC1.1 has been conceptualized for *L. racemosa* to estimate divergence time within ELR population group; **(B)** the three scenarios tested to assess the effective population size changes (ABC2). In all scenarios, t# represents time scale measured in number of generations and N# represents effective population size of the corresponding population group during the relevant time period (e.g., 0–t1, t1–t2). Abbreviations of the populations have been given in Supplementary Table S1 and the population groups have been identified in the text.

Using the cpDNA data, the demographic history of divergence between populations was assessed in the approximate Bayesian computation (ABC) framework implemented in DIYABC ver. 2.0 (Cornuet et al., 2014) following (Tomizawa et al., 2017; Yamamoto et al., 2019). We did not use the nSSR data of *L. littorea* for the ABC analysis due to presence of null alleles (see section Genetic diversity), which can potentially bias conclusions of the species’ demographic history (Thalmann et al., 2011). To keep the scenarios simple, we conceptualized two ABC models. In the first model (hereafter ABC1), seven population divergence scenarios were tested to estimate the divergence time of the population groups identified for each of the two species (Figure 2A). For *L. racemosa*, an additional simple divergence scenario (ABC1.1) was conceptualized to estimate the divergence time within the population group ELR. In the second model (hereafter ABC2), three simple population demographic scenarios were tested to examine the changes in effective population size for both species and population groups (Figure 2B). These scenarios were conceptualized as: scenario (1) constant model (Na = N1; effective population size was constant at N1 from the present to the past); scenario (2) expansion model (Na < N1; effective population size changed from Na to N1 at time t); and scenario (3) bottleneck model (Na > N1; effective population size changed from Na to N1 at time t). The default prior values were used for all parameters, including mutation rates; however, based on the results of the pilot runs, the values of the maximum population size and the maximum values of time scale were changed from default 10,000 to 100,000 to obtain better posterior distributions. One million simulations were run for each scenario and we chose the most-likely scenario based on the comparative assessment of the posterior probabilities of the scenarios. We also checked the goodness-of-fit of the selected scenario through principal component analysis (PCA). The number of haplotypes, the number of segregating sites, the mean of pairwise differences, the variance of pairwise differences, Tajima’s D, the number of private segregating sites, and the mean of numbers of the rarest nucleotide at segregating sites were used as summary statistics for each of the three population groups. The number of haplotypes, the number of segregating sites, the mean of pairwise differences, and F_ST_ were used as the summary statistics for each of the population pairs.

Mismatch distribution analyses (MDA) were performed on the cpDNA data, using ARLEQUIN, to test whether the species as a whole or the populations groups had undergone recent population expansion. The validity of the expansion model was tested using the sum of squared deviations (SSD) between the observed and expected mismatch distributions. The smoothness of the distribution curves was evaluated using Harpending’s raggedness index (H_Rag_; Harpending, 1994). In addition, D of Tajima (1989) and Fs test of Fu (1997) were also performed, using DNASP, to investigate the recent demographic changes.

To detect demographic changes in populations from each group, the nSSR data of *L. littorea* were further used to identify presence of a bottleneck event. In BOTTLENECK analysis, implemented using BOTTLENECK ver. 1.2.02 (Piry et al., 1999), both the Sign and Wilcoxon tests were applied under two microsatellite mutation models, the stepwise mutation model (SMM) and two-phased model of mutation (TPM), and the presence of mode-shift were determined.

## Results

### Haplotype Phylogeny and Divergence Time

The aligned sequences of the three chloroplast intergenic regions, *trn*T*-trn*L, *trn*G*-trn*S, and *atp*B*-rbc*L, were 490, 615, and 650 bp in *L. racemosa* and 692, 750, and 612 bp in *L. littorea*, respectively. The concatenated cpDNA sequences had a total length of 1,755 bp and 2,054 bp, with 14 variable sites (eight substitutions, four indels, and two inversions) and five variable sites (three substitutions and two indels) in *L. racemosa* and *L. littorea*, respectively (Supplementary Table S3). In combination, these polymorphisms formed four haplotypes (RH1–RH4) across the 385 samples analyzed in *L. racemosa* and three haplotypes (LH1–LH3) across the 329 samples analyzed in *L. littorea*. No haplotype was shared between the two species. Haplotype frequencies for each population are presented in Supplementary Table S4.

The chloroplast DNA phylogenetic trees constructed using Bayesian inference and Maximum-Likelihood (ML) method (Supplementary Figure S1) showed consistent topological relationships. The seven haplotypes of *Lumnitzera* sp. were clustered into two major groups corresponding to the two species. The two *Lumnitzera* sp. diverged in around 10.95 MYA (95% CI = 6.32–16.04; Supplementary Figure S1). The *L. racemosa* group was sub-divided into two clades; the haplotype RH3 was related to RH4 (clade LR-I) while RH1 was related to RH2 (clade LR-II). The *L. littorea* group was also sub-divided into two clades, showing that LH1 and LH2 (clade LL-I) were more closely related to each other than to LH3 (clade LL-II).

### Phylogeography

#### Phylogeographic Subdivision and Distribution of Haplotypes

**Figure 3:**
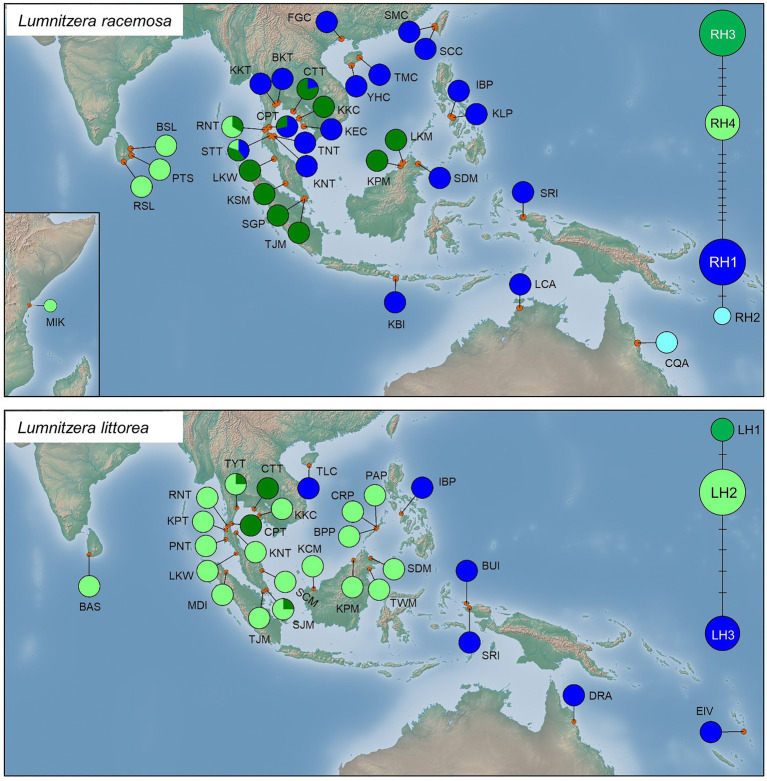
Geographical distribution of haplotypes and their frequencies within *L. racemosa* and *L. littorea* populations; median-joining network for the haplotypes in which the size of the circle is proportional to the frequency of each sampled haplotype with the branches marked indicating the number of steps separating adjacent haplotypes. Abbreviations of the populations have been given in Supplementary Table S1.

Genealogical networks and geographical distribution analyses of haplotypes also showed two similarly differentiated clades for *L. racemosa* and *L. littorea*, which showed apparent eastern vs. western geographical distributions in both species (Figure 3). In *L. racemosa*, haplotypes RH3 and RH4 formed a group (western-LR group) following the phylogenetic clade LR-I (Supplementary Figure S1), which was separated by at least nine mutational steps from the haplotypes RH1 and RH2 (eastern-LR group) which formed phylogenetic clade LR-II (Supplementary Figure S1). In *L. littorea*, haplotypes LH1 and LH2 grouped together (western-LL group), corresponding to the phylogenetic clade LL-I, which was separated by at least four mutational steps from LH3 [eastern-LL (ELL) group], corresponding to the phylogenetic clade LL-II (Supplementary Figure S1).

In *L. racemosa*, all populations from the Indian Ocean were either fixed for one of the western-LR haplotypes (four populations for RH3 and three populations for RH4) or showing a mixture of them (one population), while most populations from the Pacific Ocean were fixed for one of the eastern-LR haplotypes (16 populations for RH1 and 1 population for RH2; Figure 3). Admixture of western‐ and eastern-LR haplotypes (RH1, RH3, and/or RH4 in three populations) were observed in *L. racemosa* populations. In *L. littorea*, all seven populations from the Indian Ocean and most populations from the Pacific Ocean (14 out of 19 populations) were fixed for one of the western-LH haplotypes (17 populations for LH2 and 2 populations for LH1) or showing a mixture of them (two populations), while only five populations located in the east periphery of the Pacific Ocean were fixed for the eastern-LH haplotype (Figure 3).

#### Genetic Diversity

Haplotype diversity was estimated to be 0.606 for *L. racemosa* and 0.472 for *L. littorea*. The overall nucleotide diversity was also lower for *L. littorea* (π = 0.30 × 10^−3^) than *L. racemosa* populations (π = 0.43 × 10^−3^; Table 1). In both species, total genetic diversity H_T_ (0.625 and 0.490 for *L. racemosa* and *L. littorea*) across all sampled populations was much higher than the average intra-population diversity H_S_ (0.068 for *L. racemosa* and 0.031 for *L. littorea*), suggesting that the majority of cpDNA diversity is distributed among populations. Population differentiation was high in both *L. racemosa* (G_ST_ = 0.890; N_ST_ = 0.901) and *L. littorea* (G_ST_ = 0.937; N_ST_ = 0.981). The trend was similar at population group level for both species in which western group had higher genetic diversity and genetic differentiation than eastern group populations (Table 1). Permutation tests showed that N_ST_ was not significantly higher than G_ST_ either for the entire IWP or for the population groups separately (*p* > 0.05). Genetic diversity estimates for individual population have been given in Supplementary Table S4.

**Table 1:** Genetic diversity, population differentiation, and demographic parameters of *Lumnitzera racemosa* and *Lumnitzera littorea* based on chloroplast DNA (cpDNA) data.

Group	Diversity estimates						Mismatch distribution		Neutrality tests	
	Hd	π	H_S_	H_T_	G_ST_	N_ST_	SSD (*p* value)	H_RAG_ (*p* value)	Tajima’s D (*p* value)	Fu’s Fs (*p* value)
***L. racemosa***										
ELR1	0.098	0.001	0.065	0.097	0.331	0.391	0.011 (0.08)	0.825 (0.80)	−1.096 (0.12)	3.370 (0.92)
ELR2	0	0	--	--	--	--	--	--	--	--
WLR1	0.056	0.004	0.045	0.054	0.154	0.154	0.005 (0.07)	0.898 (0.89)	−1.835 (0.004)	2.905 (0.89)
WLR2	0.040	0.001	0.133	0.133	0	0	0.002 (0.08)	0.925 (0.86)	−1.696 (0.008)	−0.367 (0.167)
Total	0.606	0.004	0.068	0.625	0.890	0.901	0.183 (0.05)	0.369 (0.04)	4.186 (1.00)	19.585 (0.999)
***L. littorea***										
ELL	0	0	--	--	--	--	--	--	--	--
WLL1	0.043	0.003	0.044	0.052	0.151	0.151	0.001 (0.12)	0.838 (0.89)	−0.735 (0.26)	−1.231 (0.085)
WLL2	0	0	--	--	--	--	--	--	--	--
Total	0.472	0.002	0.031	0.490	0.937	0.981	0.347 (<0.001)	0.398 (0.97)	1.926 (0.98)	6.612 (0.98)

Hd, overall haplotype diversity for all populations within each region; π, nucleotide diversity; H_S_, average genetic diversity within populations; H_T_, total genetic diversity; G_ST_, interpopulation differentiation; N_ST_, the number of substitution types. The population groups have been identified in the text: (ELR1, Eastern *L. racemosa* group 1; ELR1, Eastern *L. racemosa* group 2; WLR1, Western *L. racemosa* group 1; WLR2, Western *L. racemosa* group 2; ELL1, Eastern *L. littorea* group; WLL1, Western *L. littorea* group 1; and WLL2, Western *L. littorea* group 2).

Analyzing the nSSR data of *L. littorea*, 74 alleles were found ranging from 4 to 12 alleles per locus (with an average of 6.73). The average expected (H_E_) and observed (H_O_) heterozygosity across all *L. littorea* populations were 0.416 and 0.190, respectively (Table 2). Among all populations, the highest genetic diversity was observed in CRP (N_E_ = 2.314, H_E_ = 0.504), whereas the LKW population showed the lowest genetic diversity (N_E_ = 1.360, H_E_ = 0.220). All populations except one (DRA) had positive F_IS_ values (Table 2). The Hardy-Weinberg (HW) exact test for 187 locus × population matrix (11 loci × 17 populations) revealed significant heterozygote deficiency for 116 combinations (Supplementary Table S5) whereas the global score test (*U*-test) showed heterozygote deficiency for all populations except DRA (Table 2).

**Table 2:** Genetic diversity measures for *L. littorea* populations based on nuclear microsatellite (nSSR) data.

Population	N	A_R_	N_A_	N_E_	H_O_	H_E_	F_IS_	F	PPB (%)
BAS	3	--	--	--	--	--	--	--	--
MDI	8	2.599	2.636	2.064	0.239	0.433^*^	0.465	0.438	81.82
RNT	24	1.948	2.546	1.509	0.114	0.286^*^	0.608	0.482	90.91
KPT	21	2.435	3.091	1.765	0.130	0.382^*^	0.666	0.572	100.00
LKW	7	1.909	1.909	1.360	0.078	0.220^*^	0.664	0.337	54.55
TJM	8	2.690	2.727	2.113	0.227	0.497^*^	0.560	0.554	100.00
SJM	21	2.160	2.727	1.510	0.126	0.281^*^	0.559	0.546	100.00
KNT	12	2.173	2.273	1.580	0.091	0.685^*^	0.721	0.541	72.73
CTT	13	2.657	2.818	2.158	0.224	0.471^*^	0.535	0.502	100.00
KCM	12	2.451	2.636	1.689	0.159	0.364^*^	0.573	0.599	90.91
SDM	24	2.835	3.727	1.948	0.140	0.444^*^	0.689	0.659	90.91
CRP	13	3.283	3.727	2.314	0.231	0.504^*^	0.553	0.605	100.00
PAP	21	2.345	2.636	1.772	0.178	0.354^*^	0.504	0.403	72.73
BPP	16	2.697	3.000	2.083	0.267	0.477^*^	0.448	0.441	100.00
TLC	13	2.634	2.818	2.109	0.259	0.492^*^	0.484	0.441	100.00
IBP	2	--	--	--	--	--	--	--	--
BUI	16	2.460	2.636	2.073	0.205	0.407^*^	0.505	0.313	63.64
SRI	17	2.484	2.636	1.918	0.166	0.389^*^	0.581	0.579	81.82
DRA	7	2.636	2.636	1.830	0.403	0.389	−0.039	−0.503	81.82
Mean	15	2.494	2.775	1.870	0.190	0.416	0.534	0.525	87.17

N, number of genotyped individuals in a population; A_R_, mean of allelic richness; N_A_, number of different alleles; N_E,_ effective number of alleles; I, information index; H_O,_ observed heterozygosity; H_E_, expected heterozygosity; F_IS_, inbreeding coefficient; F = fixation Index = (H_E_ ‐ Ho)/H_E_ = 1 ‐ (Ho/H_E_); and PPB, percentage of polymorphic loci.

* Indicates heterozygote deficiency (at *p* = 0.05) as revealed by the global score test (*U*-test). Matrix of Hardy-Weinberg (HW) exact test for individual locus × population has been given in Supplementary Table S5.

#### Population Structure

**Figure 4:**
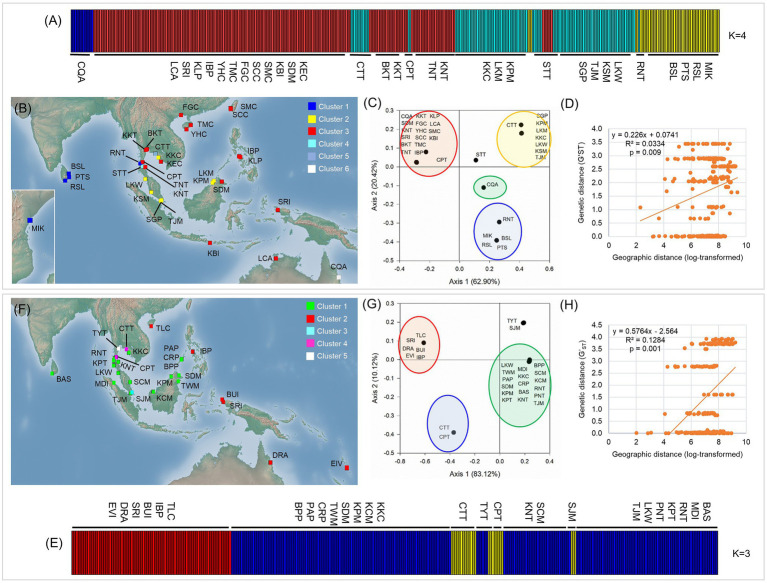
Population structure and potential barriers of gene flow in *L. racemosa* **(A–D)** and *L. littorea* **(E–H)** based on cpDNA data, as inferred from the assignment result inferred from the Bayesian Analysis of Population Structure (BAPS) analysis **(A,E)**, spatial AMOVA (SAMOVA) in which population clusters have been identified by different colors in the maps **(B,F)**, Principal Coordinate Analysis (PCoA; **C**,**G**); yellow lines in B and F representing potential biogeographic barriers identified from the Monmonier’s algorithm; scatterplots of Mantel test showing relationship between pairwise genetic and geographic distances **(D,H)**. Abbreviations of the populations have been given in Supplementary Table S1.

Bayesian Analysis of Population Structure analysis revealed that individual sampling sites of *L. racemosa* and *L. littorea* could be optimally grouped under four and three clusters, respectively. In line with the geographic distribution of the haplotypes, individuals of *L. racemosa* populations of the eastern-LR group having the RH1 haplotype formed a cluster (cluster 1) and the individuals of CQA population having the RH2 haplotype formed another cluster (cluster 2; Figure 4A). Individuals of the western-LR group also formed two clusters – populations having the RH3 haplotype formed a cluster (cluster 3) whereas those with the RH4 belonged to a separate cluster (cluster 4; Figure 4A). Genetic admixture was observed in three populations, namely STT (six individuals belonging to cluster 1, five individuals to cluster 3, and three individuals to cluster 4), CPT (five individuals belonging to cluster 1 and two individuals to cluster 3), and CTT (11 individuals belonging to cluster 3 and three individuals to cluster 1). SAMOVA analysis identified six clusters (*K* = 6 when F_CT_ = 0.939). The four clusters identified in BAPS were consistent in SAMOVA, except the admixtured populations STT and CTT belonged to two different clusters (Figure 4B). The clustering pattern from the PCoA was also nearly consistent with BAPS and SAMOVA with three genetically differentiated population clusters with two populations, namely STT and CQA, in between the clusters (Figure 4C). In case of *L. littorea*, populations of the ELL and western-LL group formed two separate clusters in BAPS analysis. Two populations of the western-LL group, namely CPT and CTT formed a separate cluster (cluster 3) with individuals from the populations of SJM and TYT (Figure 4E). The SAMOVA analysis identified five clusters (*K* = 5 when F_CT_ = 0.992) and the pattern is consistent with BAPS except the admixtured populations, namely SJM and TYT, formed two different clusters (Figure 4F). The PCoA also revealed consistent clustering pattern, except the SJM and TYT populations formed a single cluster (Figure 4G).

**Figure 5:**
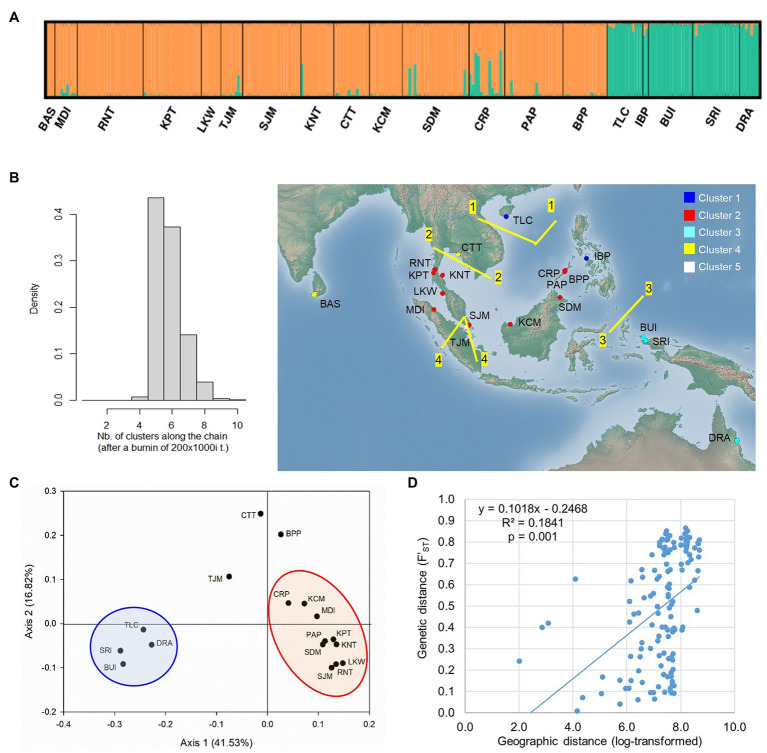
Population structure of *L. littorea* based on nSSR data as inferred from – **(A)** STRUCTURE analysis (*K* = 2), **(B)** GENELAND analysis in which population clusters (*K* = 5) have been identified by different colors in the map, **(C)** Principal Coordinate Analysis, and **(D)** scatterplots of Mantel test showing relationship between pairwise genetic and geographic distances. Abbreviations of the populations have been given in Supplementary Table S1.

The STRUCTURE analysis of nSSR loci of *L. littorea* revealed that ∆K value was maximum at the true value of *K* = 2 (Supplementary Figure S2). At *K* = 2, the average L(K) values [i.e., estimated log likelihood for each K; Ln P(D)] reached the plateau with the maximum variation between runs (Supplementary Figure S2). These findings suggest that *L. littorea* individuals can be optimally grouped under two genetic clusters (*K* = 2; Figure 5A), being consistent with the cpDNA phylogeographic structure. One cluster consisted of all individuals belonging to the populations from the Indian Ocean and most of the Pacific Ocean populations (western group). The east periphery populations (TLC, IBP, BUI, SRI, and DRA) of the Pacific Ocean belonged to another cluster (eastern group), even if multiple K values (*K* = 3, 4, 5) were considered (Supplementary Figure S2). Most individuals (247 out of 258) had >80% inferred ancestry from one genetic cluster. Considering the location information, the GENELAND analysis revealed that the populations can be optimally grouped under five clusters (*K* = 5; Figure 5B). Among the western group populations identified by the STRUCTURE analysis, BAS and CTT formed a cluster whereas TJM belonged to a separate cluster, being consistent with the pattern if higher K values of the STRUCTURE analysis were considered (Supplementary Figure S2). Among the eastern group, two populations IBP and TLC belonged to a separate cluster. The rest of the clustering pattern was found to be consistent between STRUCTURE and GENELAND analyses. In the PCoA, eastern and western group populations were separated along the *X*-axis whereas the TJM, CTT, and BPP populations were separated from the two population groups along the *Y*-axis (Figure 5C).

Based on the population structure pattern from the cpDNA data and the evolutionary relationship of the haplotypes, four population clusters were identified in *L. racemosa* – eastern-LR group (with haplotype RH1; hereafter ELR1), eastern-LR2 group (with haplotype RH2; hereafter ELR2), western-LR1 group (with haplotype RH3; hereafter WLR1), and western-LR2 group (with haplotype RH4, hereafter WLR2; Figure 2). Similarly, in *L. littorea*, three population clusters were identified – eastern-LL group (with haplotype LH3, hereafter ELL), western-LL1 group (with haplotypes LH, hereafter WLL1), and western-LL2 group (with haplotypes LH1, hereafter WLL2; Figure 2). Hierarchical AMOVA analysis of the cpDNA data revealed that majority of the variation (90.56 and 99.14% for *L. racemosa* and *L. littorea*, respectively) could be attributed to the differentiation among population clusters (Table 3). Considering each cluster independently, maximum genetic variation was found within populations in the western group. The AMOVA of the nSSR data showed that 52.07% of total genetic variation was partitioned within populations of *L. littorea* whereas 34.39% variation could be attributed to between eastern and western groups (Table 4).

**Table 3:** Analysis of molecular variance (AMOVA) for *L. racemosa* and *L. littorea* based on cpDNA data.

Source of variation	*L. racemosa*					*L. littorea*				
	df	SS	VC	PV (%)	F statistics	df	SS	VC	PV (%)	F statistics
**All populations**										
Among groups	3	862.302	3.757	90.56	F_SC_ = 0.39^*^	2	261.589	1.760	99.14	F_SC_ = 0.19^*^
Among populations	28	58.393	0.156	3.75	F_ST_ = 0.94^*^	24	1.141	0.003	0.16	F_ST_ = 0.99^*^
Within populations	353	83.243	0.236	5.68	F_CT_ = 0.91^*^	302	3.750	0.012	0.70	F_CT_ = 0.99^*^
Total	384	1003.938	4.149			328	266.480	1.775		
**ELR1**						**WLL1**				
Among populations	17	51.315	0.230	45.17	F_ST_ = 0.45^*^	18	1.141	0.004	17.57	F_ST_ = 0.176^*^
Within populations	198	55.314	0.279	54.83		210	3.750	0.018	82.43	
Total	215	106.630	0.509			228	4.891	0.022		
**WLR1**										
Among populations	7	6.137	0.047	15.02	F_ST_ = 0.15^*^					
Within populations	98	25.529	0.265	84.98						
Total	105	32.066	0.311							
**WLR2**										
Among populations	4	0.940	0.019	30.81	F_ST_ = 0.31					
Within populations	45	2.000	0.044	69.19						
Total	49	2.94	0.064							

df, degree of freedom; SS, sum of squares; VC, variance components; PV, percentage of variation; F_CT_, differentiation among regions within species; F_SC_, differentiation among populations within regions; and F_ST_, differentiation within populations. The population groups have been identified in the text (ELR1, Eastern *L. racemosa* group 1; WLR1, Western *L. racemosa* group 1; WLR2, Western *L. racemosa* group 2; and WLL1: Western *L. littorea* group 1).

* *p* < 0.001 (1,000 permutations).

**Table 4:** Analysis of molecular variance for *L. littorea* based on nSSR data.

Source of variation	df	SS	VC	PV (%)	F statistics
**All populations**					
Among populations	16	534.136	1.059	33.09	F_ST_ = 0.331^**^
Within populations	489	1047.109	2.141	66.91	
Total	505	1581.245	3.200		
**EG**					
Among populations	3	52.241	0.587	20.28	F_ST_ = 0.203^**^
Within populations	102	235.344	2.307	79.72	
Total	105	287.585	2.894		
**WG**					
Among populations	12	225.960	0.551	20.79	F_ST_ = 0.208^**^
Within populations	387	811.765	2.098	79.21	
Total	399	1037.725	2.648		
**EG vs. WG**					
Among groups	1	255.935	1.414	34.39	F_CT_ = 0.344^*^
Among populations within groups	15	278.200	0.557	13.54	F_SC_ = 0.206^**^
Within groups	489	1047.109	2.141	52.07	F_ST_ = 0.479^**^
Total	505	1581.245	4.112		

EG, Eastern Group; WG, Western Group; Population groups identified based on STRUCTURE analysis. df, degree of freedom; SS, sum of squares; VC, variance components; PV, percentage of variation; F_CT_, differentiation among regions within species; F_SC_, differentiation among populations within regions; and F_ST_, differentiation within populations.

* *p* < 0.05;

** *p* < 0.001 (1,000 permutations).

#### Determinants of Genetic Structure

The Monmonier’s algorithm based on genetic distance estimated from the cpDNA data of *L. racemosa* identified barriers between – (1) populations from the western and eastern coasts of Malay Peninsula, (2) populations from northwestern (LKM and KPM) and northeastern (SDM) Borneo, and (3) populations from western coast of Malay Peninsula and Indonesia (KBI; Figure 4B). These barriers formed a “Malay Peninsula–Borneo–Indonesia” barrier line that segregates *L. racemosa* distribution into two groups: the western group comprised of populations from the Indian Ocean and the eastern group comprised of the Pacific Ocean populations. In *L. littorea*, barriers were identified – (1) separating one South China Sea population (TLC) from the rest (populations of Gulf of Thailand, Malay Peninsula, Borneo, and Palawan), (2) between Palawan and Philippines (IBP), and (3) between populations in Borneo and Indonesian New Guinea (BUI and SRI; Figure 4F). These barriers jointly formed a “South China Sea–Mindoro Strait–Sulawesi Island–North Australia” barrier line that separated the entire IWP populations of *L. littorea* into two groups: the western group comprised of all Indian Ocean populations and most of the Pacific Ocean populations and the eastern group comprised of the east-peripheral Pacific Ocean populations. The genetic barriers identified based on the nSSR data of *L. littorea* also matched with that obtained using the cpDNA data. The Mantel test revealed significant positive relationship between geographic distance and population genetic differentiation for both species [*R*^2^ = 0.033, *p* = 0.009 for *L. racemosa* (Figure 4D); *R*^2^ = 0.128, *p* = 0.001 for *L. littorea* using cpDNA (Figure 4H); and *R*^2^ = 0.184, *p* = 0.001 using nSSR data (Figure 5D)].

#### Demographic History

In the ABC analysis (ABC1), the highest values of posterior probability were obtained for scenario 7 (0.522, 95% CI = 0.385–0.659) for *L. racemosa* and scenario 3 (0.479, 95% CI = 0.438–0.521) for *L. littorea* (Supplementary Table S6). Neither the scenarios overlapped with the 95% CI of other scenarios. Absence of significant differences between the observed and simulated data in 26 out of the 36 summary metrics (Supplementary Table S7) and the position of the observed data in close proximity of the simulated data cluster in the PCA (Supplementary Figure S3) showed that the selected scenarios were good fit for the observed data. For *L. racemosa*, the divergence times of N3 (ELR1 + ELR2) from N1 (WLR1) + N2 (WLR2), and N1 from N2 were estimated to be 69,600 (95% CI = 21,500–98,200) and 6,480 (95% CI = 744–35,900) generations ago, respectively (Supplementary Table S8). With an approximate estimate of generation time of *Lumnitzera* species around 20 years (He et al., 2018), the divergence times of were converted into absolute time of 1.39 and 0.13 MYA, respectively. From the simple ABC model, N3.1 (ELR1) split from N3.2 (ELR2) in around 46,700 (95% CI = 4,100–96,700) generations ago, corresponding to 0.93 MYA. For *L. littorea*, the divergence times of N2 (ELL) from N1 (WLL1) + N3 (WLL2), and N1 from N3 were estimated to be 84,000 (95% CI = 35,900–99,400) and 22,200 (95% CI = 3,170–74,300) generations ago, respectively (Supplementary Table S8). The divergence times of were converted into absolute time of 1.68 and 0.44 MYA, respectively.

Analyzing the changes in effective population size (ABC2), the highest value of posterior probability was found for scenario 3 (population bottleneck model) across the population groups of both species (Supplementary Table S9). No significant differences between the observed and simulated data were observed in most of the seven summary metrics (Supplementary Table S10), and the observed data were in close proximity of the simulated data cluster in the PCA (Supplementary Figure S3) for both the species and the corresponding population groups, thereby indicating that the selected scenarios were good fit for the observed data. In *L. racemosa*, the median values of t were 19,800 (95% CI = 1,280–88,800; for WLR1), 38,900 (95% CI = 3,200–95,800; for WLR2), and 16,200 (95% CI = 919–89,100; for ELR1) generations ago (Supplementary Table S8), corresponding to 0.396 (for WLR1), 0.778 (for WLR2), and 0.324 MYA (for ELR1). In *L. littorea*, the median value of t was 51,100 (95% CI = 4,940–97,400) generations ago for WLL1, corresponding to 1.022 MYA (Supplementary Table S8).

For *L. racemosa*, the mismatch distribution for all populations was multimodal (Supplementary Figure S4), and neutrality tests of Tajima’s D and Fu’s Fs statistics also did not support the population expansion hypothesis (Table 1; Supplementary Table S4). When the population groups were considered, the WLR1 and WLR2 populations showed unimodal distributions (Supplementary Figure S4), non-significant SSD and H_Rag_ (both *p* > 0.05), and significantly negative Tajima’s D, however with positive and non-significant Fu’s Fs (Table 1). No compelling evidence of recent expansion was also found for ELR1 (significant SSD, non-significant Tajima’s D, and positive Fu’s Fs). Mismatch distribution analysis for all populations of *L. littorea* showed a unimodal graph (Supplementary Figure S4), which was statistically consistent with the expansion model in terms of H_Rag_ (*p* > 0.05) but not of SSD (*p* < 0.05). The neutrality tests also showed positive Tajima’s D and Fu’s Fs values (Table 1; Supplementary Table S4). When analyzed separately for the population groups, although statistical tests of both SSD and H_Rag_ and negative Tajima’s D and Fu’s Fs values (although non-significant) could explain the expansion model, the observed curve for the WLL1 populations was not of a typical unimodal distribution (Supplementary Figure S4), thereby finding no strong support for population expansion of *L. littorea* in the IWP.

Based on the nSSR data of *L. littorea*, the more conservative Wilcoxon test of the bottleneck analysis revealed that only two populations (SJM and BUI) deviated from mutation-drift equilibrium (Supplementary Table S11). Thirteen out of 17 populations had normal L-shaped distribution, which suggested that majority of *L. littorea* populations did not experience recent bottleneck.

## Discussion

Our study revealed that the two *Lumnitzera* species diverged in around 10.95 MYA, corresponding to the Late Oligocene to Early Miocene, the timeframe characterized with rapid northern movement of the Australia plate and its collision with the Sunda Shelf (now the Malay Peninsula). Previous studies have found co-occurrence of the hybrid species *Lumnitzera* × *rosea* with the parent *L. racemosa* and *L. littorea* mostly along the eastern coast of the Malay Peninsula (Guo et al., 2011; Duke, 2017), suggesting re-association of the diversified parental lineages. These evidence indicate that the geological and climate changes induced by this continental drift leading to vicariance and re-association might influence the speciation process in *Lumnitzera*, as observed in other mangrove taxa (e.g., in *Pelliciera* in the Atlantic East Pacific, Duke, 2020). Our study identified the biogeographic factors that might have shaped the present distribution of genetic diversity and population structure of *L. racemosa* and *L. littorea* across their distribution range. Although the basal genetic divergence was mostly concordant between the marker types, marked difference in population structure was observed between the two species.

### Genetic Diversity

At the species level, genetic diversity estimates of *L. littorea* (H_T_ = 0.490) and *L. racemosa* (H_T_ = 0.625) were much lower compared to other mangrove species studied using cpDNA markers, e.g., another non-viviparous *Excoecaria agallocha* (H_T_ = 0.740; Guo et al., 2018a) and cryptoviviparous mangrove *Avicennia germinans* (H_T_ = 0.87; Nettel and Dodd, 2007). At the population level, we also found low genetic diversity for both species. Only two out of 27 populations of *L. littorea* and four out of 32 populations of *L. racemosa* were found to be polymorphic. The positive Tajima’s D and Fu’s Fs values for all polymorphic populations indicate the presence of an excess of intermediate frequency alleles which can result from population bottlenecks, structure, and/or balancing selection (Biswas and Akey, 2006). The ABC analysis of effective population size changes supported the bottleneck model for both species and across all population groups. The estimated time of population size changes indicates that during Pleistocene sea-level changes, *Lumnitzera* populations experienced steep bottlenecks which might lead to loss of genetic diversity at the population level (Saenger, 1998). Similar paucity of genetic diversity due to bottleneck events has been observed in other mangrove species such as *Sonneratia alba* (Yang et al., 2017) and *Avicennia marina* (Maguire et al., 2000).

The low genetic diversity may also be attributed to the mating system of the species. Previous studies have shown that pollinator-mediated self-pollination in *L. racemosa* could contribute to a high selfing rate in this self-compatible species (Aluri et al., 2014). Indeed, we found more than 90% variation residing within populations of *L. racemosa* in the IWP. *Lumnitzera littorea*, on the other hand, is pollinated by birds (Zhang et al., 2017), which tends to be more effective than insect-mediated pollination (Cronk and Ojeda, 2008) and encourages outcrossing. However, the species suffers from low pollen viability and efficiency, heavy embryo abortion, and insect-mediated damage in reproductive period (Zhang et al., 2017). Analysis of nSSR data of *L. littorea* also showed heterozygote deficiency thereby indicating the inbreeding influence, although the heterozygote deficiency can also be caused by presence of null alleles or the Wahlund effect (Chun et al., 2010). We found positive inbreeding values for most of the populations; however, the presence of null alleles at microsatellite loci can influence the F_IS_ estimates (Chapuis and Estoup, 2006). Indeed, out of 11 nSSR loci, 3–5 loci showed presence of null alleles in all but one populations of *L. littorea*. Rather, we found a strong population structure with minimum genetic exchange in between and isolation of populations in small fragments with more than 80% variation among them. In general, the majority of the total genetic diversity resided within populations in case of outcrossing species, whereas more genetic diversity is proportioned among populations in selfing species (Hamrick and Godt, 1990). The high genetic variation among population of both the species indicate that low genetic diversity in *Lumnitzera* sp. might arise from population substructure or founder events, in which a high level of inbreeding might lead to creation of demes of genetically similar individuals.

### Population Structure

Although such local diversity may occasionally be inflated by genetic admixture from other differentiated populations, we found little evidence of genetic exchange between geographically and genetically distinct populations of *L. littorea* and *L. racemosa*. Rather, we identified strong population structure from the genetic clustering algorithms which showed, more or less unanimously, that the distributional range of both *Lumnitzera* species can be divided into population groups with different haplotype composition and phylogenetic differentiation. The AMOVA indicated that maximum proportion of variation (>70%) could be attributed to differentiation among these population groups. However, while both species had the eastern-western clustering pattern, the boundary for the clusters was more eastward for *L. littorea* compared to *L. racemosa*, signifying that the demographic forces could differently influence population structuring of the congeneric species.

#### Population Structure in *L. racemosa*

The geographic distribution of the *L. racemosa* haplotypes and the clustering algorithms revealed that populations from the Indian Ocean and the Pacific Ocean belonged to two highly divergent groups, as found in many other mangrove species of this region (e.g., *Rhizophora*, Ng et al., 2015; *Bruguiera*, Urashi et al., 2013). The divergence time between the haplotypes of these two regions corresponded to Early Pleistocene (in around 1.39 MYA), an epoch characterized with changes in oceanic circulation pattern and climate in Sundaland (Lohman et al., 2011). Previous studies using haplotypes of the nuclear genes indicated that divergence of the two lineages happened as a result of multiple glaciations and continued until Early-Middle Pleistocene (Li et al., 2016), as also observed in the divergence times of populations within the Indian and Pacific Oceanic regions in this study. The emergence of the Malay Peninsula as a part of the Sundaland at this time cut off genetic exchange between the Indian and Pacific Oceans. In our study, the Monmonier’s algorithm also identified the Malay Peninsula as one of the geographic barriers in this region, suggesting its role in impediment of genetic exchange across the two oceanic regions.

Another possible reason for such restricted dispersal could be the buoyancy period and longevity of *L. racemosa* propagules. Previous field experiments have shown that the predominant buoyancy pattern of *L. racemosa* propagules can be categorized as a sinker (i.e., sink after shedding the pericarp) and the propagules have very low (15 days) buoyancy period (Clarke et al., 2001). Therefore, it is logical to assume that the species cannot travel a long distance across the open ocean like other mangrove species having large diaspores with buoyancy period of several months (e.g., *Heritiera littoralis* and *Rhizophora* species; Van Der Stocken et al., 2019). The IBD analysis also showed significant positive relationship between geographic distance and genetic differentiation in *L. racemosa* across the IWP.

The haplotype similarity between the populations of the South East Asia and northern Australia could be traced back to the Late Miocene when the Australian plate was nearer to the Sundaland margin (Hall, 2009), thereby facilitating genetic exchange between these two regions. Present day, LDD-assisted gene flow appears to be unlikely given the presence of Sahul Shelf as the land barrier (Lohman et al., 2011), the cryptic barrier generated due to the Indonesia ThroughFlow (Banerjee et al., 2020), and the restricted LDD ability of the *L. racemosa* propagules (Clarke et al., 2001). Genetic differentiation across the Indo-Australian Archipelago in many mangrove taxa has been found to be caused by these factors (Urashi et al., 2013; Duke, 2017). The ABC analysis indicated that haplotype divergence between populations of north (LCA) and east (CQA) coasts of Australia could have happened during Middle Pleistocene (in around 0.93 MYA), probably because of the species’ restricted distribution in Pleistocene refugia in east Australia, the presence of which has been reported for numerous other plant taxa (Hilbert et al., 2007; Das et al., 2019).

#### Population Structure in *L. littorea*

The clustering pattern of *L. littorea* based on both cpDNA and nSSR data, however, suggested insignificant barrier effect of the Malay Peninsula. Sharing the same haplotype between populations of the Indian and Pacific Oceans suggests that the Malay Peninsula might not be an effective dispersal barrier for *L. littorea*. This can be explained based on the high dispersal ability of the species’ propagules with floating and viability periods of 214 days (Steele, 2006). Rather, pronounced genetic differentiation in *L. littorea* was observed along another biogeographic barrier, the Huxley’s line. The Huxley’s line is a known biogeographic boundary that runs through the Lombok Strait in the south and northward between Sulawesi and Borneo, extending further between the Palawan Island and the rest of the present Philippine islands (Simpson, 1977). While initially based on evidences from birds, the Huxley’s line has also been found to act as a phytogeographic boundary (Van Welzen et al., 2011; Duke, 2017).

The divergence time estimate indicated that these haplotypes (LH3 from LH1+LH2) diverged in Late Pliocene (in around 1.68 MYA), suggesting a possible vicariant history caused by isolation, as observed in many plant and animal taxa (Lohman et al., 2011). Further loss of suitable habitats and environments during the glacial phases of the Pleistocene might have restricted the species to the refugial populations near the equator. Such populations which persisted throughout glacial maxima in refugia are often characterized with higher genetic diversity and/or spatially patterned genetic differentiation (Provan and Bennett, 2008). The genetic diversity estimates indicated possible presence of such refugia along the east coast of the Malay Peninsula (haplotype diversity) and in around the Philippine islands (nuclear genetic diversity). The presence of glacial refugia in these locations has also been found in other studies (Cannon et al., 2009).

Although the ELL populations were clustered differently from the WLL populations in the GENELAND analysis based on the nSSR data, the position of the biogeographic barriers differed between the marker types. No barrier was observed between the populations of Philippines (IBP) and Palawan (CRP, BPP, and PAP). The STRUCTURE analysis also revealed genetic admixture in Palawan populations, suggesting nuclear gene flow through dispersal of pollen grains between these two regions. Similar asymmetrical mobility between pollen and seed gene flow leading to discordance of phylogeographic structure has been reported for other species as well (Bai et al., 2010). The nSSR data further identified a dispersal barrier between populations of South East Asia and Australia, which were clustered differently in the GENELAND analysis. These findings suggest that similar to *L. racemosa*, haplotype sharing between the ELL populations was unlikely to be assisted by present-day LDD of *L. littorea* propagules. The South Equatorial Counter Current of the Indonesian Throughflow (ITF) could facilitate propagule dispersal between the east coast of Australia and New Guinea, however, further northward dispersal gets hindered due to presence of the Halmahera Eddy (Hall, 2009), thereby acting as a cryptic barrier to gene flow.

### Conservation Implications

Our study revealed low genetic diversity of *Lumnitzera* species compared to other mangrove species of this region and a strong population structure with restricted genetic exchange in between the geographic regions. The haplotype diversity was centered in a few geographic areas of the species’ distribution range. Further, high amount of genetic variation among populations of the two species indicated high level of inbreeding within genetically similar individuals present in fragmented demes. In line with the observation for other mangroves, fragile habitat, and environmental conditions as well as anthropogenic impacts are continuously threatening the survival of these species. Indeed, *L. littorea* has been found to become endangered in many areas of its distribution range. For example, the species can only be found in the limited areas in the nature reserve in Hainan Island of China. Habitat fragmentation and isolation of population coupled with poor reproductive capacity in *L. littorea*, and high amount of selfing and restricted dispersal ability of *L. racemosa* are threatening the species’ survival in near future. In this context, the genetic information retrieved from this study can provide some conservation insights.

High genetic variation among populations in both species indicated that each population should be conserved separately. Populations with high genetic diversity, e.g., SJM, TYT of *L. littorea* and RNT, STT, CPT, and CTT of *L. racemosa* should be prioritized for conservation actions. Restoring the disturbed habitats, implementing community-based conservation programs, and long-term monitoring of genetically isolated populations could be effective strategies to maintain genetic diversity and prevent irrevocable genetic erosion in these areas. Besides, long-term *in situ* conservation programs, like creating and maintaining natural reserves, are necessary and monitoring of natural populations can be effective conservation strategies for populations with both high and low genetic diversity. Due to the impediment of genetic exchange by the natural barriers, translocations among the genetically isolated units might be a feasible potion to supplement locally depleted and/or extinct natural resource (Hu et al., 2017). However, caution should be exercised while introducing foreign germplasms as it may disrupt local adaptation, spread deleterious alleles or cause outbreeding depression (Su et al., 2010). Therefore, transfer of germplasms between the populations belonging to the same genetic group (e.g., between the east coast of the Malay Peninsula and China) should be attempted to increase their probability of survival. Our study indicated that the east coast of the Malay Peninsula and Philippines might constitute a glacial refugium during the Pleistocene epoch. The importance of conserving genetic diversity for historically survived relic populations has long been acknowledged (Frankham, 2005) and therefore, this region should be prioritized for conservation actions. Finally, similar to the *Lumnitzera* species, low genetic diversity and strong population structure have been found for other mangroves inhabiting the IWP region (Guo et al., 2018c). The conservation actions, when implemented, may minimize human influence and habitat loss, thereby can benefit other marine taxa, and maintain an integrated coastal ecosystem functioning.

## Conclusion

Our study revealed relatively low genetic diversity and prominent population structure in both *L. racemosa* and *L. littorea*. The position of genetic break was found to vary between the two species, indicating that the two *Lumnitzera* species have different phylogeographic patterns despite their close genetic relationship and similar current geographic distribution. It is important to note here that variable number of sample sizes might have affected the estimated diversity parameters (Leberg, 2002). However, fairly equal number of individuals sampled across most of the sites, very low polymorphism, and considering the variation of sample sizes in the estimation of diversity parameters might minimize the biasness generated from the sampling size variation. The ABC analysis is a powerful tool and has been widely used to infer population divergence time for other marine taxa (e.g., Tomizawa et al., 2017; Yamamoto et al., 2019), the estimates might suffer from uncertainties like generation time of the species, overlapping of generations, and 95% CI of the inferred parameters (Tsuda et al., 2015). However, given the high genetic differentiation and limited evidence of haplotype sharing between populations of distinct regions, the bias of temporal estimates might be limited. Since the population split occurred before the Last Glacial Maximum (ca. 20,000 years BP), the effect of ignoring post-divergence gene flow on divergence times between populations should also be minimum here.

## Data Availability Statement

The representative sequences of cpDNA haplotypes and SSR loci have been deposited in Genbank under the accession numbers MW244372-MW244392 and MT274028-MT274038, respectively.

## Author Contributions

WG and YH conceived the idea and designed the study. WG, HW, SQ, HF, and YL collected the samples and data. WG, AB, and HW analyzed the data and interpreted the results. WG, AB, HW, WN, and YH wrote the manuscript text. All authors reviewed the manuscript and have approved the final version of the article.

### Conflict of Interest

The authors declare that the research was conducted in the absence of any commercial or financial relationships that could be construed as a potential conflict of interest.
