# Supplementary material for: Contrasting Phylogeographic Patterns in Lumnitzera Mangroves Across the Indo-West Pacific
Source: Front Plant Sci. 2021 Jun 23;12:637009. doi: 10.3389/fpls.2021.637009 (PMC8261646; doi:10.3389/fpls.2021.637009)

Table S1. Sampling details for *Lumnitzera racemosa* and *Lumnitzera littorea*

Country	Population code	Location	Longitude, Latitude	Sample size		Collectors and collection dates
				cpDNA	nSSR	
L. racemosa						
Kenya	MIK	Mida Creek	39.9501,-3.3667	9	-	S. He, 2017/02
Sri Lanka	RSL	Rekawa	80.8500,6.0500	12	-	Y. Liu & Y. Huang, 2016/10
	PTS	Pottuvil	81.8167,6.8667	12	-	Y. Liu & Y. Huang, 2016/10
	BSL	Batticaloa	81.7000,7.7167	14	-	Y. Liu & Y. Huang, 2016/10
Thailand	RNT	Ranong	98.6667,10.1167	3	-	C. Jaengjai, 2009/01
	STT	Surat Thani	99.2686,9.3708	15	-	Y. Huang, 2016/07
	KNT	Khanom	99.8651,9.2373	8	-	Y. Huang, 2016/07
	TNT	Thong Nian Bay	99.8010,9.3002	13	-	X. Li, 2010/08
	CPT	Chumphon	99.2333,10.4500	7	-	Y. Huang, 2016/07
	KKT	Klong Kone	99.9734,13.3262	8	-	C. Jaengjai, 2009/01
	BKT	Bangkok	100.2764,13.5093	12	-	C. Jaengjai, 2012/01
	CTT	Chanthaburi	102.2667,12.4333	14	-	C. Jaengjai, 2012/01
Malaysia	LKW	Langkawi	99.8258,6.4370	18	-	Y. Liu & Y. Huang, 2013/11
	KSM	Kuala Selangor	101.2500,3.3333	8	-	Y. Liu & Y. Huang, 2013/11
	KPM	Kuala Penyu	115.7984,5.5343	16	-	Y. Liu & Y. Huang, 2013/11
	LKM	Likas	116.1002,5.9923	9	-	Y. Liu & Y. Huang, 2013/11
	TJM	Tanjung Piai, Johor	103.5000,1.2667	14	-	S. Qiao & S. He, 2013/11
	SDM	Sandakan	118.0073,5.8175	12	-	S. Qiao & S. He, 2013/11
Singapore	SGP	Singapore	103.6935,1.4024	9	-	Y. Huang, 2016/07
Cambodia	KKC	Koh Kong	102.9833,11.5500	18	-	T. Tang, 2014/02
	KEC	Kep	103.5660,10.5394	11	-	T. Tang, 2014/02
Indonesia	KBI	Bali	115.1862,-8.7436	14	-	S. He, 2017/07
	SRI	Sorong	131.2851,-0.8963	10	-	S. Shi, 2012/11
China	SMC	Shengmumiao, Taiwan	120.0986,23.2307	13	-	S. Shi, 2004/10
	SCC	Sicao, Tainan,Taiwan	120.1493,23.0148	12	-	S. Shi, 2004/10
	FGC	Fangchenggang, Guangxi	108.3333,21.6000	14	-	S. He, 2014/07
	TMC	Tanmengang, Hainan	110.6263,19.2427	13	-	S. He & Y. Huang, 2013/05
	YHC	Yalongwan, Hainan	109.6202,18.2204	13	-	S. He & Y. Huang, 2013/05

Philippines	IBP	Ibajay	122.1141,11.8247	8	-	S.He, 2013/07
	KLP	Kalibo	122.4188,11.6451	6	-	S. He, 2013/07
Australia	LCA	Ludmilla Creek, Darwin	130.8388,-12.4126	27	-	Y. Huang, 2018/03
	CQA	Cairns	145.7502,-16.8667	13	-	Y. Huang, 2018/03
L. littorea						
Sri Lanka	BAS	Madu Ganga River	80.0511,6.2833	5	3	Y. Liu & Y. Huang, 2016/10
Indonesia	MDI	Medan	98.5346,3.9482	8	8	S. Shi, 2011/12
	BUI	Raja Ampat, Papua	130.9054,-0.3045	16	16	S. Shi, 2012/11
	SRI	Sorong	131.2841,-0.8959	18	17	S. Shi, 2012/11
Thailand	RNT	Ranong	98.6667,10.1167	16	24	C. Jaengjai, 2009/01
	KPT	Bang Ben, Kapoe	98.4599,9.5815	18	21	X. Li, 2010/08
	PNT	Phang-Nga Bay	98.4753,8.3351	7	-	Y. Huang, 2016/07
	KNT	Khanom	99.8503,9.2167	12	12	Y. Huang, 2016/07
	CPT	Chumphon	99.2333,10.4502	6	-	Y. Huang, 2016/07
	TYT	Phetchaburi	99.9555,12.5929	8	-	C. Jaengjai, 2009/01
	CTT	Chanthaburi	102.2667,12.4333	13	13	C. Jaengjai, 2009/01
Malaysia	LKW	Langkawi	99.8651,6.4146	10	7	Y. Liu & Y. Huang, 2013/11
	TJM	Tanjung Piai National Park	103.5091,1.2668	8	8	S. Qiao & S. He, 2013/11
	SJM	Sg. Johor	103.9833,1.5167	12	21	Y. Liu & Y. Huang, 2013/11
	SCM	Sg. Cherating	103.3833,4.1167	13	-	S. Qiao & S. He, 2013/11
	KCM	Kuching	110.3167,1.6667	23	12	Y. Liu & Y. Huang, 2013/11
	KPM	Kuala Penyu	115.6615,5.5373	18	-	Y. Liu & Y. Huang, 2013/11
	SDM	Sandakan	118.0063,5.8187	15	24	S. Qiao & S. He, 2013/11
	TWM	Tawau	117.8179,4.3057	9	-	Y. Liu & Y. Huang, 2013/11
Cambodia	KKC	Koh Kong	102.9833,11.5503	8	-	T. Tang, 2014/02
Philippines	CRP	Calatubog River, Palawan	118.7506,9.9333	13	13	S. He, 2014/02
	PAP	Nacidoc, Palawan	118.8167,9.9503	10	21	S. He, 2014/02
	BPP	Puerto Princesa City	118.7197,9.7793	16	16	S. He, 2014/02
	IBP	Ibajay, Aklan	122.1065,11.8277	6	2	S. He, 2013/07
China	TLC	Tielugang, Hainan	109.6931,18.2642	13	13	S. He & Y. Huang, 2013/05
Australia	DRA	Daintree River	145.3697,-16.2605	12	7	S. Shi, 2014/02
Vanuatu	EIV	Efate island	168.2428,-17.5936	16	-	S. Shi, 2014/02

Table S2. Sequences of the primers used for amplification of three non-coding cpDNA regions and 11 nSSR loci

**CpDNA primers for *L. racemosa* and *L. littorea***				
Primer pairs	Primer sequences 5'→3'	Ta (^{0}C)	References	
*atp*B	GTGGAAACCCCGGGACGAGAAGTAGT	55	(Hodges and Arnold, 1994)	
*rbc*L	ACTTGCTTTAGTTTCTGTTTGTGGTGA			
*trn*S(GCU)	GCCGCTTTAGTCCACTCAGC	60	(Hamilton, 1999)	
*trn*G(UCC)	GAACGAATCACACTTTTACCAC			
*Unit*A	CATTACAAATGCGATGCTCT	60	(Taberlet et al., 1991)	
*Unit*B	TCTACCGATTTCGCCATATC			
**SSR primers for *L. littorea***				
Primer pairs	Primer sequences 5'→3'	Allele size (bp)	Repeat motif	Ta (^{0}C)
LL-144-F	GGCTCTACTCAACGCCTGTC	191	(CAC)5	53
LL-144-R	TGGACACAGAGGGAACATCA			
LL-379-F	ATGAGCACTCTCCCATCCAC	258	(AG)8	53
LL-379-R	CCTTCCTTCTAACGTGAGCG			
LL-852-F	GGGGTTAGAGTGGATGCAGA	224	(TC)6	53
LL-852-R	TTCTCATCGCCAGTTCACAG			
LL-1086-F	CACTTTTCCCGACAACCTGT	273	(CCA)6	53
LL-1086-R	GGAGTGATTGAGGTCGGAAA			
LL-1150-F	TGATGTGAGAGTTCTTGCGG	242	(TC)8	53
LL-1150-R	TGCCCTCTGTTCACTTCCTC			
LL-2749-F	GTCCCTCTCCCAAAAGGAAG	159	(TGC)5	53
LL-2749-R	CACTCGAGCATGAGGTTTCA			
LL-2952-F	AATTCCAGGCCACTCCTTCT	271	(AAG)5	53
LL-2952-R	CAACGTGACCAGCTTCTTCA			
LL-3258-F	CAGCCTAGCATGTCTGGTGA	199	(AT)7	53
LL-3258-R	GCCTTTTCTTTTTCGATCTGC			
LL-3573-F	CGCTTAGCTGCCTCCATTAC	215	(GA)9	53
LL-3573-R	CGAGCGAAAATTGGATTCAT			
LL-4058-F	TCGGTGTTCATATGCATCGT	217	(GGT)7	53
LL-4058-R	CCGCTACTGCTGGTGTCATA			
LL-4164-F	AGCTGAAGCTGAAGCTGAGG	265	(AAT)7	53
LL-4164-R	GCACCTCGACGTTTCTTCTC			

Ta = annealing temperature

References

Hamilton, M.B. (1999). Four primer pairs for the amplification of chloroplast intergenic regions with intraspecific variation. *Molecular ecology* 8**,** 521-523.

Hodges, S.A., and Arnold, M.L. (1994). Columbines: a geographically widespread species flock. *Proceedings of the National Academy of Sciences* 91**,** 5129-5132.

Taberlet, P., Gielly, L., Pautou, G., and Bouvet, J. (1991). Universal primers for amplification of three non-coding regions of chloroplast DNA. *Plant Molecular Biology* 17**,** 1105-1109.

Table S3. Variable sites of the aligned sequences of four haplotypes of *Lumnitzera racemosa* and three haplotypes of *Lumnitzera littorea*

L. racemosa

	*trn*T-L spacer			*trn*G-S spacer						*atp*B-*rbc*L spacer				
	75-79	110	151	211	254-255	262	443-488	558	561	36	94-114	135	488-500	631
RH1	--	A	A	A	TC	T	b	C	A	G	c	T	--	C
RH2	--	A	A	C	TC	T	b	C	A	G	c	T	--	C
RH3	a	T	G	A	GA	G	--	A	C	T	--	G	d	A
RH4	a	T	G	A	TC	T	--	A	C	T	c	G	--	A
RH1	--	A	A	A	TC	T	b	C	A	G	c	T	--	C

a: ACTAT; b: GACTAAATAAGAAGATTATATTAGACTAAATAAGAAGATTATATTA;
c: CTAATATTATCAACTAATTAA; d: AGATAATATCTAT

L. littorea

	*trn*T-L spacer	*trn*G-S spacer			*atp*B-*rbc*L spacer
	111-124	38	231-234	453	89-93
LH1	a	C	b	G	d
LH2	--	C	b	G	d
LH3	--	G	c	T	--

a: ATAATAGCTATTAA; b: TTTC; c: GAAA; d: CTAAT; --: indel

Table S4. Genetic diversity estimates for *Lumnitzera racemosa* and *Lumnitzera littorea* based on cpDNA data

Population	H	S	Hd	π (e^{-3})	T-D	F-F
L. racemosa						
MIK	RH4(9)	0	0.000	0.000	NA	NA
RSL	RH4(12)	0	0.000	0.000	NA	NA
PTS	RH4(12)	0	0.000	0.000	NA	NA
BSL	RH4(14)	0	0.000	0.000	NA	NA
RNT	RH3(1), RH4(2)	4	0.667	1.516	1.641	3.022
STT	RH1(6), RH3(6), RH4(3)	13	0.686	3.813	1.497	11.780
KNT	RH1(8)	0	0.000	0.000	NA	NA
TNT	RH1(13)	0	0.000	0.000	NA	NA
CPT	RH1(5), RH3(2)	13	0.476	3.526	1.599	9.891
KKT	RH1(8)	0	0.000	0.000	NA	NA
BKT	RH1(12)	0	0.000	0.000	NA	NA
CTT	RH1(3), RH3(11)	13	0.363	2.688	1.203	11.379
LKW	RH3(18)	0	0.000	0.000	NA	NA
KSM	RH3(8)	0	0.000	0.000	NA	NA
KPM	RH3(16)	0	0.000	0.000	NA	NA
LKM	RH3(9)	0	0.000	0.000	NA	NA
TJM	RH3(14)	0	0.000	0.000	NA	NA
SDM	RH1(12)	0	0.000	0.000	NA	NA
SGP	RH3(9)	0	0.000	0.000	NA	NA
KKC	RH3(18)	0	0.000	0.000	NA	NA
KEC	RH1(11)	0	0.000	0.000	NA	NA
KBI	RH1(14)	0	0.000	0.000	NA	NA
SRI	RH1(10)	0	0.000	0.000	NA	NA
SMC	RH1(13)	0	0.000	0.000	NA	NA
SCC	RH1(12)	0	0.000	0.000	NA	NA
FGC	RH1(14)	0	0.000	0.000	NA	NA
TMC	RH1(13)	0	0.000	0.000	NA	NA
YHC	RH1(13)	0	0.000	0.000	NA	NA
IBP	RH1(8)	0	0.000	0.000	NA	NA
KLP	RH1(6)	0	0.000	0.000	NA	NA
LCA	RH1(27)	0	0.000	0.000	NA	NA
CQA	RH2(13)	0	0.000	0.000	NA	NA
L. littorea						
BAS	LH2(5)	0	0.000	0.000	NA	NA
MDI	LH2(8)	0	0.000	0.000	NA	NA
BUI	LH3(16)	0	0.000	0.000	NA	NA
SRI	LH3(18)	0	0.000	0.000	NA	NA
RNT	LH2(16)	0	0.000	0.000	NA	NA
KPT	LH2(18)	0	0.000	0.000	NA	NA
PNT	LH2(7)	0	0.000	0.000	NA	NA
KNT	LH2(13)	0	0.000	0.000	NA	NA
CPT	LH1(6)	0	0.000	0.000	NA	NA
TYT	LH1(2), LH2(6)	1	0.429	0.21	0.334	0.536
CTT	LH1(13)	0	0.000	0.000	NA	NA
LKW	LH2(10)	0	0.000	0.000	NA	NA
TJM	LH2(8)	0	0.000	0.000	NA	NA
SJM	LH1(3), LH2(9)	1	0.409	0.200	0.541	0.735

SCM	LH2(13)	0	0.000	0.000	NA	NA
KCM	LH2(23)	0	0.000	0.000	NA	NA
KPM	LH2(18)	0	0.000	0.000	NA	NA
SDM	LH2(15)	0	0.000	0.000	NA	NA
TWM	LH2(9)	0	0.000	0.000	NA	NA
KKC	LH2(8)	0	0.000	0.000	NA	NA
CRP	LH2(13)	0	0.000	0.000	NA	NA
PAP	LH2(10)	0	0.000	0.000	NA	NA
BPP	LH2(16)	0	0.000	0.000	NA	NA
IBP	LH3(6)	0	0.000	0.000	NA	NA
TLC	LH3(13)	0	0.000	0.000	NA	NA
DRA	LH3(12)	0	0.000	0.000	NA	NA
EIV	LH3(16)	0	0.000	0.000	NA	NA

H, chlorotype (count); S, number of segregating sites; Hd, haplotype diversity; π, nucleotide diversity; T-D, Tajima's D; F-F, Fu's Fs

Table S5. Matrix of locus (11) x population (17) of *Lumnitzera littorea* showing p-values of the Hardy-Weinberg (HW) exact test against the hypothesis of heterozygote deficiency

	Locus										
Populations	L1	L2	L3	L4	L5	L6	L7	L8	L9	L10	L11
MDI	NA	0.0008	0.0351	1.0000	0.0155	0.0314	0.0416	0.0154	0.0676	0.0024	NA
RNT	<0.001	NA	0.0000	1.0000	0.0211	0.0017	0.0012	0.0002	0.0000	0.1109	1.0000
KPT	<0.001	0.0242	<0.001	1.0000	0.0005	<0.001	0.0312	<0.001	0.0006	0.3789	1.0000
LKW	NA	NA	0.0768	0.0771	NA	0.0025	0.0774	NA	0.0765	1.0000	NA
TJM	0.0157	0.0666	0.0226	1.0000	0.0150	0.0803	0.1242	0.0068	0.0868	0.3839	0.0074
SJM	<0.001	0.0019	0.0001	1.0000	0.0243	0.0245	0.0092	<0.001	0.0243	0.7033	1.0000
KNT	0.0022	0.0016	0.0065	NA	0.0007	0.0017	0.0881	0.0061	NA	0.0094	NA
CTT	0.0005	0.0006	0.0932	1.0000	0.0051	0.0203	<0.001	0.0054	0.0005	0.0005	1.0000
KCM	0.0022	0.0440	0.0002	NA	0.0017	1.0000	0.0622	0.0062	0.0022	0.1305	0.1292
SDM	<0.001	<0.001	<0.001	NA	0.0008	<0.001	<0.001	<0.001	0.0002	0.2987	<0.001
CRP	0.0399	0.0388	0.0039	0.0013	0.0001	0.0011	0.0004	0.0003	0.5274	0.0419	0.0001
PAP	0.0001	0.0018	0.2245	NA	NA	0.0027	0.0008	NA	<0.001	0.0025	0.0009
BPP	0.0002	0.1900	0.0090	1.0000	0.0003	0.0804	0.4813	0.0004	0.0001	0.0004	0.0004
TLC	0.0012	0.0001	0.0065	0.0753	<0.001	0.0018	1.0000	0.0002	0.0019	1.0000	1.0000
BUI	NA	<0.001	0.0003	NA	0.0002	0.0019	0.0087	<0.001	NA	0.0006	NA
SRI	NA	<0.001	0.0002	1.0000	0.0005	0.0002	0.0044	<0.001	0.0304	0.0109	NA
DRA	1.0000	NA	0.1019	0.0366	0.0776	0.4352	0.0392	NA	1.0000	0.0196	0.0497

NA: Locus monomorphic; no test done

Values marked in red indicate significance at p<0.05

Abbreviations of the populations have been given in Table S1

Table S6. Posterior probability of each of the seven scenarios (ABC1 model) for *Lumnitzera racemosa* and *Lumnitzera littorea*, and their 95% confidence interval based on the logistic estimate

Species	Scenario	Posterior probability	95% Confidence Interval (lower, upper)
L. racemosa	1	0.0320	0.0000,0.2234
	2	0.0093	0.0000,0.2089
	3	0.0284	0.0000,0.2210
	4	0.0401	0.0000,0.3135
	5	0.2016	0.0538,0.3494
	6	0.1664	0.0027,0.3300
	7	0.5222	0.3854,0.6590
L. littorea	1	0.0900	0.0213,0.1587
	2	0.0269	0.0000,0.1068
	3	0.4794	0.4381,0.5207
	4	0.0255	0.0000,0.1057
	5	0.0169	0.0000,0.1266
	6	0.3398	0.3039,0.3757
	7	0.0214	0.0000,0.1026

Table S7. Numerical results of model checking (ABC1 model) in terms of one sample and two sample summary statistics for *Lumnitzera racemosa* and *Lumnitzera littorea*

Summary statistics		Observed value			Proportion (simulated<observed)		
		L. racemosa		*L. littorea*	*L. racemosa*		*L. littorea*
One sample summary statistics	NHA_1_1	2	3	2	0.385	0.2115	0.439
	NHA_1_2	2	1	1	0.1405	0.264	0.293
	NHA_1_3	4		1	0.5055		0.266
	NSS_1_1	3	11	1	0.7585	0.853	0.43
	NSS_1_2	11	0	0	0.979*	0.264	0.293
	NSS_1_3	12		0	0.9815*		0.266
	MPD_1_1	0.12	0.9919	0.0429	0.378	0.593	0.4125
	MPD_1_2	0.6108	0	0	0.611	0.264	0.293
	MPD_1_3	1.0456		0	0.788		0.266
	VPD_1_1	0.3459	9.267	0.0411	0.682	0.945	0.411
	VPD_1_2	6.3467	0	0	0.975*	0.264	0.293
	VPD_1_3	8.8211		0	0.985*		0.266
	DTA_1_1	-1.696	-1.0959	-0.7347	0.005*	0.11	0.2085
	DTA_1_2	-1.8353	0	0	0*	0.513	0.536
	DTA_1_3	-1.1414		0	0.099		0.541
	PSS_1_1	0	11	1	0.123	0.8625	0.45
	PSS_1_2	0	0	0	0.1145	0.2755	0.295
	PSS_1_3	1		0	0.324		0.288
	MNS_1_1	1	10.1818	5	0.273	0.264	0.4505
	MNS_1_2	3	0	0	0.214	0.264	0.293
	MNS_1_3	10.4167		0	0.337		0.266
Two sample summary statistics	NH2_1_1&2	3	4	3	0.112	0.205	0.3825
	NH2_1_1&3	4	12	2	0.228	0.7665	0.276
	NH2_1_2&3	4		2	0.249		0.2735
	NS2_1_1&2	11		5	0.907		0.7005
	NS2_1_1&3	12		1	0.9325		0.2675
	NS2_1_2&3	12		5	0.9525*		0.8085
	MP2_1_1&2	0.5222	0.9886	0.0382	0.5475	0.593	0.334
	MP2_1_1&3	1.0041		0.0426	0.7815		0.408
	MP2_1_2&3	0.9692		0	0.766		0.1635
	MPB_1_1&2	3.0849	1.5185	4.0218	0.88	0.426	0.8755
	MPB_1_1&3	7.8331		0.9782	0.992*		0.71
	MPB_1_2&3	10.284		5	0.999*		0.9215
	HST_1_1&2	0.8307	0.349	0.9905	0.8635	0.4235	0.819
	HST_1_1&3	0.8718		0.9564	0.8095		0.8215
	HST_1_2&3	0.9058		1	1*		0.8945

NHA: Number of haplotypes; NSS: Number of segregating sites; MPD: Mean of pairwise differences; VPD: Variance of pairwise differences; DTA: Tajima's D; PSS: Private segregating sites; MNS: Mean of numbers of the rarest nucleotide at segregating sites

NH2: Number of haplotypes; NS2: Number of segregating sites; MP2: Mean of pairwise differences (W); MPB: Mean od pairwise differences (B); HST: Fst following Hudson et al. 1992

Table S8. Estimated divergence parameters for the population groups of *Lumnitzera racemosa* and *Lumnitzera littorea*

Species	Model	Parameters	Mean	Median	Mode	95% Confidence Interval (lower-upper)
L. racemosa	ABC1	N1	43900	40400	27900	7270-93500
		N2	77900	82400	88700	34000-98800
		N3	62700	65800	76500	15600-95500
		t1	9530	6480	2270	744-35900
		t2	67300	69600	94100	21500-98200
		NA	69800	76000	96200	11400-98900
	ABC1.1	N1	43000	39000	26600	4190-95200
		N2	15800	8770	150	256-72800
		t1	48500	46700	21600	4100-96700
		NA	57700	61300	88100	4300-98200
L. littorea	ABC1	N1	25800	20400	7620	2090-76800
		N2	13500	7960	2230	613-59300
		N3	21300	13700	3080	1240-77500
		t1	26700	22200	10600	3170-74300
		t2	79700	84000	99400	35900-99400
		NA	62700	67800	98400	6170-98700
L. racemosa	ABC2	N1	15400	8210	1450	841-67600
		t	21600	11000	941	516-88800
		NA	68200	73700	98000	13700-99000
WLR1		N1	5250	2000	434	218-33000
		t	28100	19800	2710	1280-88800
		NA	71200	76400	98600	20800-99000
WLR2		N1	19300	12100	3530	1620-70100
		t	43200	38900	16000	3200-95800
		NA	61100	64400	97900	11400-98500
ELR1		N1	13200	7620	4010	1410-56700
		t	25500	16200	2520	919-89100
		NA	69800	74600	93300	20100-99000
L. littorea	ABC2	N1	18600	12300	4030	1730-67000
		t	40000	34600	6960	1530-95800
		NA	60400	63200	87100	11300-98100
WLL1		N1	26200	8210	1310	472-99000
		t	51300	51100	51200	4940-97400
		NA	49000	48600	11600	2860-97400

The population groups have been identified in the text: (ELR1: Eastern *Lumnitzera racemosa* group 1; WLR1: Western *Lumnitzera racemosa* group 1; WLR2: Western *Lumnitzera racemosa* group 2; WLL1: Western *Lumnitzera littorea* group 1)

Table S9. Posterior probability and their 95% confidence interval of each of the three scenarios (ABC2 model) for *Lumnitzera racemosa* and *Lumnitzera littorea*, and the population groups to evaluate effective population size changes

Species	Scenario	Posterior probability	95% Confidence Interval (lower, upper)
L. racemosa	1	0.3434	0.3266,0.3601
	2	0.0181	0.0090,0.0272
	3	0.6385	0.6217,0.6553
WLR1	1	0.3251	0.2799.0.3702
	2	0.0663	0.0416,0.0911
	3	0.6086	0.5617,0.6556
WLR2	1	0.3168	0.2954,0.3382
	2	0.1783	0.1611,0.1956
	3	0.5049	0.4820,0.5277
ELR1	1	0.3159	0.2771,0.3546
	2	0.0389	0.0208,0.0570
	3	0.6452	0.6061,0.6843
L. littorea	1	0.3580	0.3363,0.3797
	2	0.0825	0.0681,0.0969
	3	0.5595	0.5376,0.5813
WLL1	1	0.3220	0.3125,0.3315
	2	0.1649	0.1570,0.1727
	3	0.5131	0.5031,0.5232

The population groups have been identified in the text: (ELR1: Eastern *Lumnitzera racemosa* group 1; WLR1: Western *Lumnitzera racemosa* group 1; WLR2: Western *Lumnitzera racemosa* group 2; WLL1: Western *Lumnitzera littorea* group 1)

Table S10. Numerical results of model checking (ABC2 model) in terms of one sample summary statistics for *Lumnitzera racemosa* and *Lumnitzera littorea*, and the population groups to evaluate effective population size changes

Species	Summary statistics	Observed value	Proportion (simulated<observed)
L. racemosa	NHA_1_1	4	0.425
	NSS_1_1	12	0.7645
	MPD_1_1	5.2288	0.916
	VPD_1_1	24.4802	0.907
	DTA_1_1	4.186	0.97*
	PSS_1_1	12	0.7645
	MNS_1_1	138.4167	0.948
WLR1	NHA_1_1	2	0.7425
	NSS_1_1	11	0.9775*
	MPD_1_1	0.6108	0.889
	VPD_1_1	6.3467	0.975*
	DTA_1_1	-1.8353	0.002*
	PSS_1_1	11	0.9775*
	MNS_1_1	3	0.7015
WLR2	NHA_1_1	2	0.258
	NSS_1_1	3	0.5545
	MPD_1_1	0.12	0.2535
	VPD_1_1	0.3459	0.4855
	DTA_1_1	-1.696	0.017*
	PSS_1_1	3	0.5545
	MNS_1_1	1	0.1765
ELR1	NHA_1_1	3	0.3265
	NSS_1_1	11	0.8815
	MPD_1_1	0.9919	0.6395
	VPD_1_1	9.267	0.892
	DTA_1_1	-1.0959	0.0825
	PSS_1_1	11	0.8815
	MNS_1_1	10.1818	0.286
L. littorea	NHA_1_1	3	0.2225
	NSS_1_1	5	0.567
	MPD_1_1	1.6249	0.827
	VPD_1_1	3.7406	0.889
	DTA_1_1	1.9262	0.925
	PSS_1_1	5	0.567
	MNS_1_1	69.6	0.87
WLL1	NHA_1_1	2	0.427
	NSS_1_1	1	0.4145
	MPD_1_1	0.0429	0.4195
	VPD_1_1	0.0411	0.4195
	DTA_1_1	-0.7347	0.2255
	PSS_1_1	1	0.4145
	MNS_1_1	5	0.4525

The population groups have been identified in the text: (ELR1: Eastern *Lumnitzera racemosa* group 1; WLR1: Western *Lumnitzera racemosa* group 1; WLR2: Western *Lumnitzera racemosa* group 2; WLL1: Western *Lumnitzera littorea* group 1)

Table S11. Results of bottleneck analyses for 17 populations of *Lumnitzera littorea*

Population	TPM		SMM		Mode shift
	Sign test	Wilcoxon test (2-tailed)	Sign test	Wilcoxon test (2-tailed)	
MDI	0.169	0.426	0.182	0.496	Shifted
RNT	0.585	0.695	0.333	0.375	L-shaped
KPT	0.614	0.365	0.578	0.32	L-shaped
LKW	0.249	0.156	0.204	0.109	L-shaped
TJM	0.066	0.067	0.133	0.175	Shifted
SJM	0.079	**0.016**	**0.002**	**0.005**	L-shaped
KNT	0.229	0.742	0.504	1.000	L-shaped
CTT	0.316	0.123	0.392	0.32	Shifted
KCM	0.249	0.492	0.122	0.322	L-shaped
SDM	0.545	0.557	0.079	0.160	L-shaped
CRP	0.557	0.520	0.490	0.413	L-shaped
PAP	0.526	0.461	0.211	0.383	L-shaped
BPP	0.388	0.240	0.606	0.638	L-shaped
TLC	**0.041**	0.102	0.056	0.320	L-shaped
BUI	**0.019**	**0.008**	0.379	**0.039**	L-shaped
SRI	0.131	0.203	0.171	0.496	Shifted
DRA	0.353	0.652	0.306	0.301	L-shaped

TPM, Two phased model of mutation; SMM, Somatic model of mutation
Bold entries indicate significance ($p < 0.05$) in Sign and Wilcoxon tests

Figure S1. Phylogenetic trees constructed using concatenated sequences of three cpDNA loci. The phylogenetic trees were constructed using - (A) BEAST ver. 2.5 under the star-beast mode. Posterior support ratios were showed in grey. The estimated divergence time and 95% confidence interval for deep nodes given by MCMC simulations was shown in color. (B) RAxML-NG ver. 0.9.0 under the GTRCAT model. Supporting ratios were obtained by 1000 bootstrapping replicates and shown on nodes. (C) MRBAYES ver. 3.2.7 under the GTRGAMMA model. Posterior probability are showed for nodes.

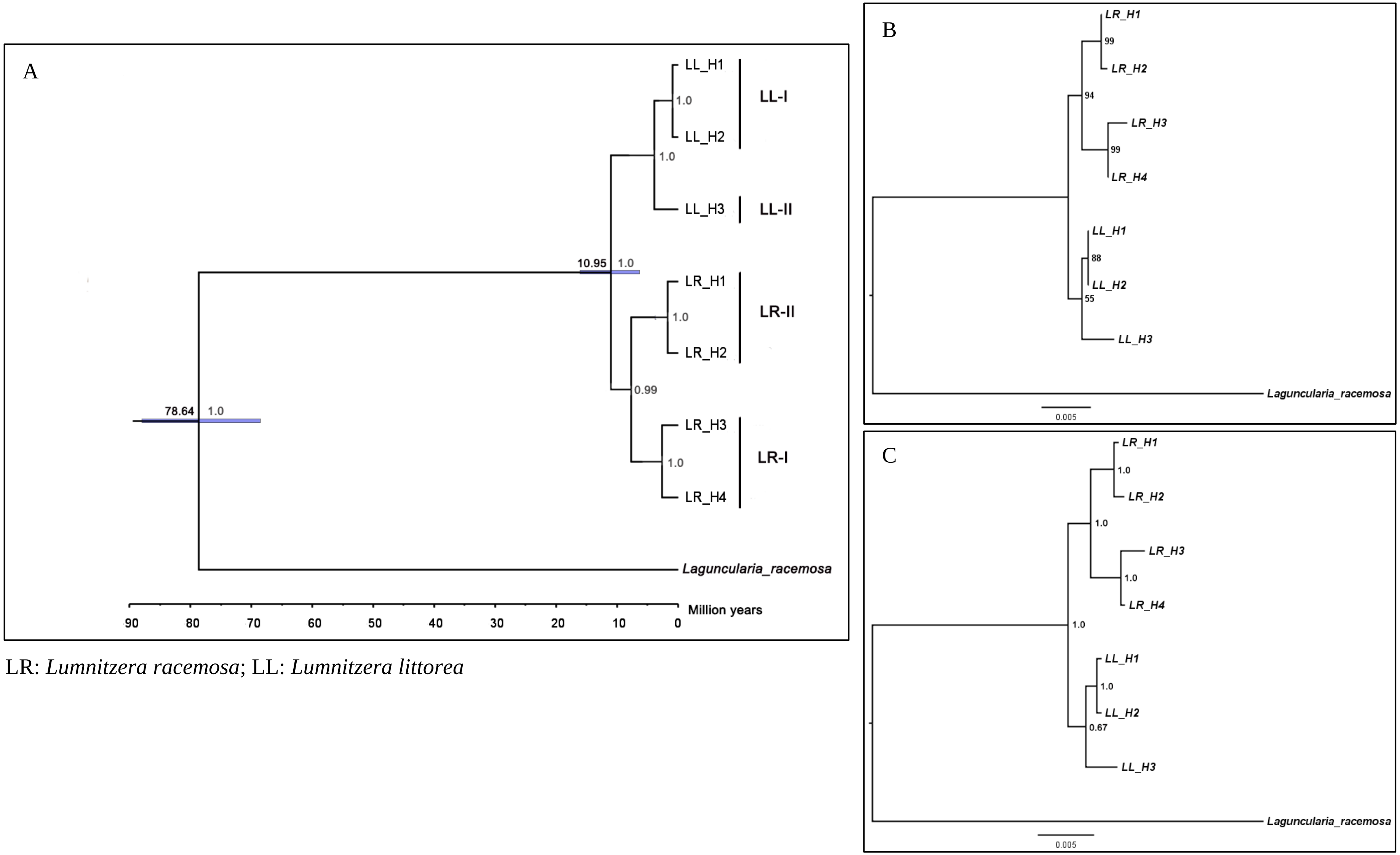

LR: *Lumnitzera racemosa*; LL: *Lumnitzera littorea*

Figure S2. Results of the STRUCTURE analysis of nSSR data of *Lumnitzera littorea* – A) the Delta K values for the true values of K; B) mean of estimated probability for each K with the error bars representing the standard deviation, C) population structure at four different K values.

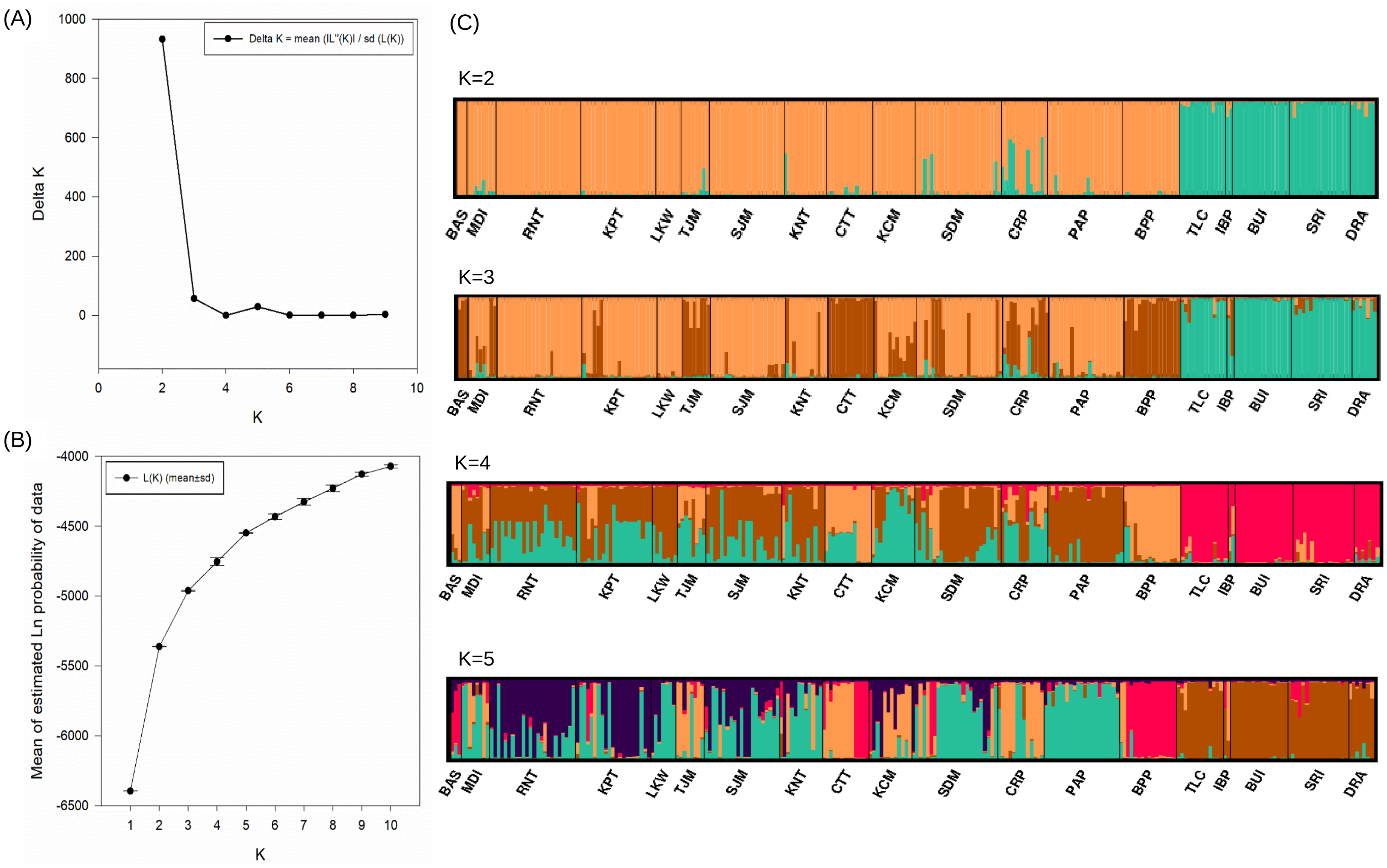

Figure S3. Principal Component Analysis (PCA) of "model checking" for A) divergence time estimation and B) assessing population size changes in *Lumnitzera racemosa* and *Lumnitzera littorea*

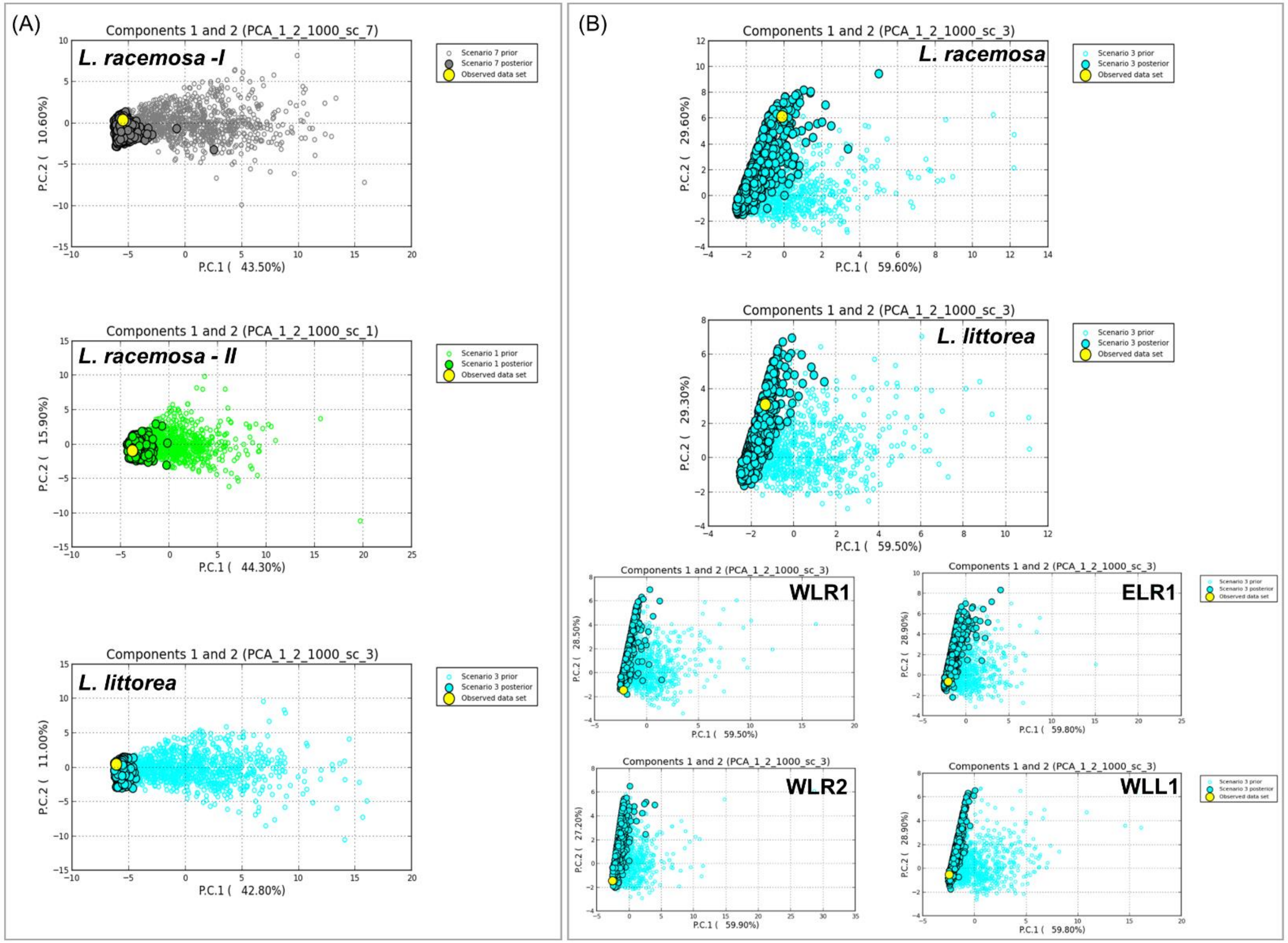

Figure S4. Mismatch distribution analysis for *Lumnitzera racemosa* and *Lumnitzera littorea*

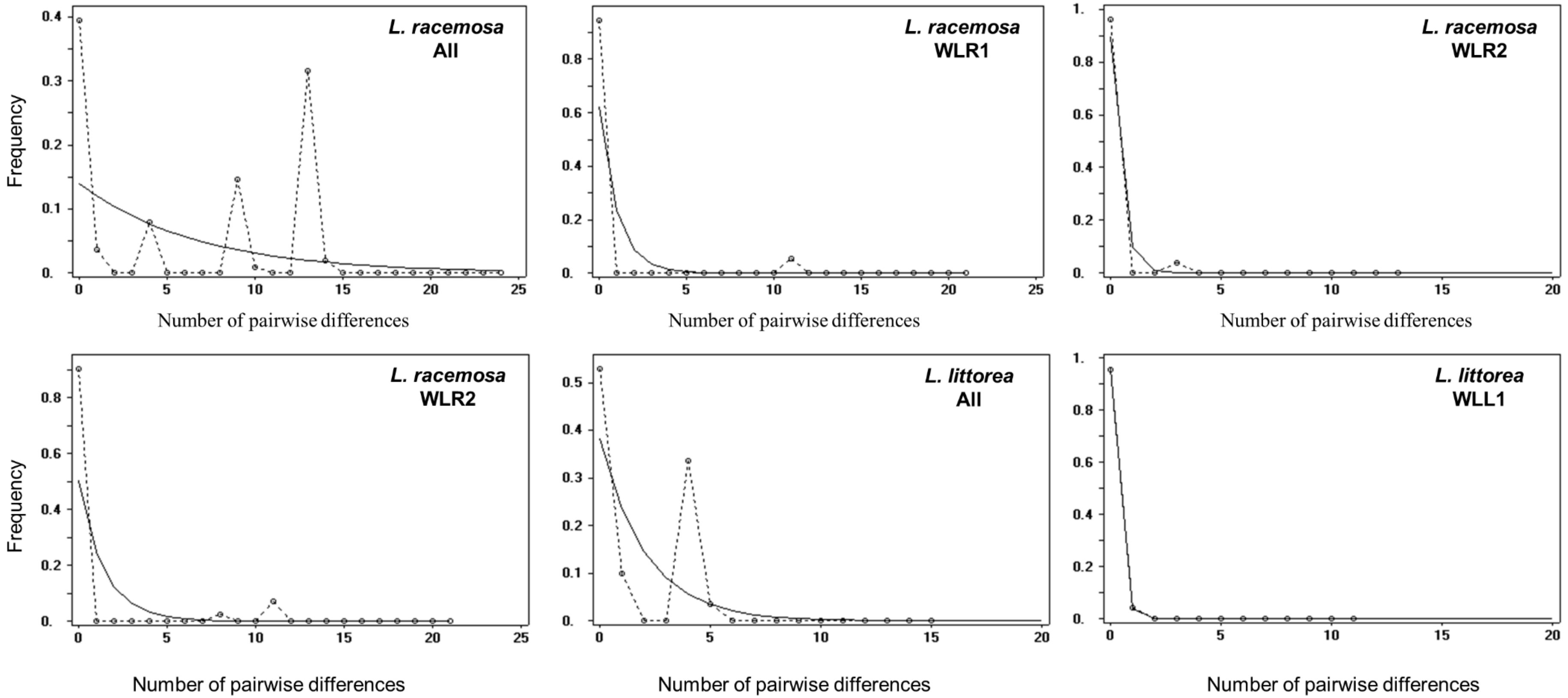

Supplement: Supplementary file 1 [file Data_Sheet_1.PDF]
